# A numerical and analytical study of SE(Is)(Ih)AR epidemic fractional order COVID-19 model

**DOI:** 10.1186/s13662-021-03447-0

**Published:** 2021-06-15

**Authors:** Hasib Khan, Razia Begum, Thabet Abdeljawad, M. Motawi Khashan

**Affiliations:** 1 Department of Mathematics, Shaheed Benazir Bhutto University, Sheringal, Dir Upper, Khyber Pakhtunkhwa Pakistan; 2 Department of Mathematics and General Sciences, Prince Sultan University, Riyadh, Saudi Arabia; 3 Department of Medical Research, China Medical University, Taichung, Taiwan; 4 Department of Computer Science and Information Engineering, Asia University, Taichung, Taiwan; 5 Department of Basic Sciences, Common First Year, King Saud University, Riyadh, 11451 Saudi Arabia

**Keywords:** Fractal fractional derivatives, Existence and uniqueness of the solutions, Hyers–Ulam stability, Numerical scheme

## Abstract

This article describes the corona virus spread in a population under certain assumptions with the help of a fractional order mathematical model. The fractional order derivative is the well-known fractal fractional operator. We have given the existence results and numerical simulations with the help of the given data in the literature. Our results show similar behavior as the classical order ones. This characteristic shows the applicability and usefulness of the derivative and our numerical scheme.

## Introduction

The end of year 2019 was shocking for the world, especially for Chinese people in Wuhan city where a novel corona virus (COVID-19) was identified with rapid transmission rate. Later this virus spread in almost all parts of the globe at pandemic level and caused 111 million infections with 2.6 million deaths. According to Johns Hopkins University, the biggest amount of cases were reported in the United States of America with a tally of 28.1 M infections and 497 K deaths. Initially, it was considered that this virus came from the local fish market in Wuhan city; however, the transmission was identified from people to people with a huge ratio. This transmission happened through water, food, air droplets, and through physical contact with an infected person. The symptoms of COVID-19 infection last for 14 days, and to overcome or to resist the spread of this infection, 20 seconds of hands wash, avoidance of social gathering, and wearing face masks was suggested by the World Health Organization (WHO). Many countries banned traveling of people from one place to another to minimize the spreading ratio and also defined policies which can uplift the balance between country economy and health sector [1]. The scientists analyzed and made different experiments to find the cure or any medicinal treatment of the COVID-19 infection. Different countries have endorsed various mitigation strategies; however, the world still awaits the arrival of vaccine which is the only tool to fight against this infection. Approximately, 100 vaccines are under development, and some of these are in Phase 3 stage of clinical trials [2]. The number of vaccines has been identified in this regard with different recovery percentage. Currently, three vaccines are authorized and recommended to prevent COVID-19 i.e. Pfizer-BioNTech, Moderna’s, and China’s Sinopharm COVID-19 vaccine. As of December 28, 2020, large-scale (Phase 3) clinical trials have been in progress or being planned for three COVID-19 vaccines in the United States, namely AstraZeneca’s COVID-19 vaccine, Janssen’s COVID-19 vaccine, and Novavax’s COVID-19 vaccine [3]. Recently, scientists from the field of medical engineering acknowledged the importance of mathematical modeling of any pandemic disease. Many of such mathematical modeling examples have already contributed to the control of infections [4–7]. These models also can be used for the prediction of expected patients in the future and can define well the control strategies. The mathematical models are usually developed in ordinary (ODEs) or in partial differential equations (PDEs) having equations of integration of natural order (IDEs). Such types of equations are well utilized in various fields of science i.e. medicine, economic, business, engineering, and analysis of different infections [8–16]. Recently, the implementation and application of fractional calculus for different models got attention from researchers [17–20]. Fractional calculus is defined as various kinds of possibilities of defining real or complex number powers [21–23]. Fractional calculus of any disease model plays a vital role in making decisions and helping to control the spread of infections. The fractional calculus was first communicated between Leibnitz and L’Hospital for the nth derivative of *y*. Fractional derivative was first introduced by Lacroix [24]. Afterward, many of the researchers introduced fractional derivatives in different forms, among which the most valuable are Caputo fractional derivative [25], Riemann–Liouville fractional derivative [26], and Atangana–Baleanu derivative [27]. Recently, various models have been solved by using fractional differential equations in many fields such as dynamics, control theory, and biology. The existence, uniqueness, and stability of models have been studied deeply [28–33]. In recent research a new idea of differentiation i.e. that the operator has fractional order as well as fractal dimension if the operator is of order two was proposed [34]. Usually, nonlinear models need specific parameters which are not available from experiments. The possible solution of these problems has been addressed by using fractal fractional derivatives. The fractal fractional derivatives models have advantage over the standard integer order derivatives [35, 36].

We use fractal fractional deactivate for the following formulation of SE(Is)(Ih)AR epidemic model with the help of [37]:

1 $$ \textstyle\begin{cases} {}^{FF} {}_{0}D^{u_{1},u_{2}}_{\tau } S(\tau )= b_{1} -[b_{2}+ \beta (I_{s}+ \beta _{hr}I_{h}+ \beta _{ar}A )+K_{v}]S+\eta R, \\ {}^{FF} {}_{0}D^{u_{1},u_{2}}_{\tau } E(\tau )=-(b_{2}+\gamma )E+ \beta (I_{s}+\beta _{hr}I_{h}+\beta _{(}ar)A)S, \\ {}^{FF} {}_{0}D^{u_{1},u_{2}}_{\tau } I_{s}(\tau )=-(b_{2}+ \tau _{0})I_{s}+ \gamma P_{s} E, \\ {}^{FF} {}_{0}D^{u_{1},u_{2}}_{\tau } I_{h}(\tau )=-(b_{2}+\alpha + \tau _{0}+K_{T}) I_{h}+\gamma P_{h}E, \\ {}^{FF} {}_{0}D^{u_{1},u_{2}}_{\tau } A(\tau )=-(b_{2}+ \tau _{0})A + \gamma (1-P_{s}-P_{h})E, \\ {}^{FF} {}_{0}D^{u_{1},u_{2}}_{\tau } R(\tau )=-(b_{2}+\eta )R+ \tau _{0}(I_{s}+I_{h}+A)+K_{T} I_{h}+K_{v}S, \end{cases} $$

where $t> 0$ with the initial conditions $S(0) = S_{0}$, $E(0) = E_{0}$, $I_{s}(0) = I_{s}(0)$, $I_{h}(0) = I_{h}(0)$, $A(0) = A_{0}$,and $R(0) = R_{0}$ subject to $\min (S_{0},E_{0}, I_{s_{0}}, I_{h_{0}},A_{0},R_{0}) \geq 0$. It is clear that the dimensions of both sides of the model are equivalent. In this model, $b_{1}$ is the recruitment rate, $b_{2}$ is the natural average death rate, $\beta (t)$, $\beta _{hr}\beta (t)$, $\beta _{ar}\beta (t)$ are the rates of transmission to the susceptible, $\frac{1}{\eta }$ is the average time of transition from the recovered to the susceptibles, *γ* is the rate of transition from the exposed class to the infectious group, *α* is the average mortality of the symptomatic infectious population, $\tau _{0}$ is the natural immune response rate for the infected people, $p_{s}, p_{h}, p_{a} = 1-p_{s}-p_{h}$ are the fractions of the exposed that become slightly symptomatic, seriously symptomatic, and asymptomatic infected people, respectively.

We highlight some more related articles used for the definitions and applications of the following notions [38–51].

### Definition 1.1

Suppose that *ψτ* is a continuous function and fractal differentiable in the interval $(a,b)$ of order $u_{2}$, then the fractal fractional derivative of *ψτ* of order $u_{1}\in (0,1)$ in the Caputo sense is given by

2 $$ {}^{FF}{}_{0}D^{u_{1},u_{2}}_{\tau }\psi (\tau )= \frac{AB(u_{1})}{1-u_{1}} \int _{0}^{\tau }\frac{d}{dt^{u_{2}}}E_{u_{1}} \biggl(-\frac{u_{1}}{1-u_{1}}(\tau -s)^{u_{1}} \biggr) \psi (s)\,ds,$$

where $AB(u_{1})=1-u_{1}+\frac{u_{1}}{\Gamma u_{1}}$.

### Definition 1.2

Suppose that $\psi (\tau )$ is a continuous function in the interval (a, b), then the fractal fractional integral of $\psi (\tau )$ of order $u_{1}$ having a Mittag-Leffler type kernel is given by

3 $$ {}^{FF} {}_{0}I^{u_{1},u_{2}}_{\tau }\psi (\tau )= \frac{u_{1} u_{2}}{AB(u_{1})\Gamma u_{1}} \int _{0}^{\tau }s^{u_{2}-1} \psi (s) (\tau -s)^{u_{1}-1}\,ds+ \frac{u_{2}(1-u_{1})\tau ^{u_{2}-1}}{AB(u_{1})}\psi (\tau ). $$

## Existence criteria

With the help of fixed point procedure we check the existence of fractal fractional to SE(Is)(Ih)AR epidemic model (1). We have

4 $$ \textstyle\begin{cases} S(t)-S(0) \\ \quad =\frac{u_{1} u_{2}}{AB(u_{1})\Gamma u_{1}} \int _{0}^{t} s^{u_{2}-1}(t-s)^{u_{1}-1}(b_{1} -[b_{2}+ \beta (I_{s}+\beta _{hr}I_{h}+ \beta _{ar}A )+K_{v}]S+\eta R)\,ds \\ \qquad {}+\frac{u_{2}(1-u_{1})t^{u_{2}-1}}{AB(u_{1})}(b_{1} -[b_{2}+ \beta (I_{s}+ \beta _{hr}I_{h}+ \beta _{ar}A )+K_{v}]S+\eta R), \\ E(t)-E (0) \\ \quad =\frac{u_{1} u_{2}}{AB(u_{1})\Gamma u_{1}} \int _{0}^{t} s^{u_{2}-1}(t-s)^{u_{1}-1}(-(b_{2}+ \gamma )E+\beta (I_{s}+\beta _{hr}I_{h}+\beta _{ar}A)S)\,ds \\ \qquad {}+\frac{u_{2}(1-u_{1})t^{u_{2}-1}}{AB(u_{1})}(-(b_{2}+\gamma )E+\beta (I_{s}+ \beta _{hr}I_{h}+\beta _{ar}A)S), \\ I_{s}(t)-I_{s} (0)=\frac{u_{1} u_{2}}{AB(u_{1})\Gamma u_{1}} \int _{0}^{t} s^{u_{2}-1}(t-s)^{u_{1}-1}(-(b_{2}+ \tau _{0})I_{s}+\gamma P_{s} E)\,ds \\ \hphantom{I_{s}(t)-I_{s} (0)=}{}+\frac{u_{2}(1-u_{1})t^{u_{2}-1}}{AB(u_{1})}(-(b_{2}+ \tau _{0})I_{s}+ \gamma P_{s} E), \\ I_{h}(t)-I_{h} (0)=\frac{u_{1} u_{2}}{AB(u_{1})\Gamma u_{1}} \int _{0}^{t} s^{u_{2}-1}(t-s)^{u_{1}-1}(-(b_{2}+\alpha + \tau _{0}+K_{T}) I_{h}+ \gamma P_{h}E)\,ds \\ \hphantom{I_{h}(t)-I_{h} (0)=}{}+\frac{u_{2}(1-u_{1})t^{u_{2}-1}}{AB(u_{1})}(-(b_{2}+\alpha + \tau _{0}+K_{T}) I_{h}+\gamma P_{h}E), \\ A(t)-A (0)=\frac{u_{1} u_{2}}{AB(u_{1})\Gamma u_{1}} \int _{0}^{t} s^{u_{2}-1}(t-s)^{u_{1}-1}(-(b_{2}+ \tau _{0})A + \gamma (1-P_{s}-P_{h})E)\,ds \\ \hphantom{A(t)-A (0)=}{}+\frac{u_{2}(1-u_{1})t^{u_{2}-1}}{AB(u_{1})}(-(b_{2}+ \tau _{0})A + \gamma (1-P_{s}-P_{h})E), \\ R(t)-R (0) \\ \quad =\frac{u_{1} u_{2}}{AB(u_{1})\Gamma u_{1}} \int _{0}^{t} s^{u_{2}-1}(t-s)^{u_{1}-1}(-(b_{2}+ \eta )R+ \tau _{0}(I_{s}+I_{h}+A)+K_{T} I_{h}+K_{v}S)\,ds \\ \qquad {}+\frac{u_{2}(1-u_{1})t^{u_{2}-1}}{AB(u_{1})}(-(b_{2}+\eta )R+ \tau _{0}(I_{s}+I_{h}+A)+K_{T} I_{h}+K_{v}S). \end{cases} $$

Now, we define some functions $Q_{i}$ and some constants $\eta _{i}$, $i\epsilon N_{1}^{6}$ as follows:

5 $$ \textstyle\begin{cases} Q_{1}(t,S)= b_{1} -[b_{2}+ \beta (I_{s}+\beta _{hr}I_{h}+ \beta _{ar}A )+K_{v}]S+\eta R, \\ Q_{2}(t,E)=-(b_{2}+\gamma )E+\beta (I_{s}+\beta _{hr}I_{h}+\beta _{ar}A)S, \\ Q_{3}(t,I_{s})=-(b_{2}+ \tau _{0})I_{s}+\gamma P_{s} E, \\ Q_{4}(t,I_{h})==-(b_{2}+\alpha + \tau _{0}+K_{T}) I_{h}+\gamma P_{h}E, \\ Q_{5}(t,A)=-(b_{2}+ \tau _{0})A + \gamma (1-P_{s}-P_{h})E, \\ Q_{6}(t,R)=-(b_{2}+\eta )R+ \tau _{0}(I_{s}+I_{h}+A)+K_{T} I_{h}+K_{v}S. \end{cases} $$

(G*): For proving our results, we assume the following assumptions: The continuous functions $S(t)$, $E(t)$, $I_{s}(t)$, $I_{h}(t)$, $A(t)$, $R(t)$ and $S^{*}(t)$, $E^{*}(t)$, $I_{s}^{*}(t)$, $I_{h}^{*}(t)$, $A^{*}(t)$, $R ^{*}(t)$ all belong to $L[0,1]$ such that $\|I_{s}\|\leq \psi _{1}$, $\|I_{h}\|\leq \psi _{2}$, $\|A\|\leq \psi _{3}$ for $\psi _{1}$, $\phi _{2}$, $\psi _{3}> 0$ and constants.

### Theorem 2.1

*The kernels* $Q_{i}$ *for* $i=1,2,3,\ldots,6$ *satisfy Lipschitz conditions if the assumption* (G*) *holds and satisfies* $\phi _{i}< 1$ *for* $i\in N_{1}^{6}$.

### Proof

First, we prove that $Q_{1}(t, S)$ satisfies the Lipschitz condition. Using $S(t)$, $S^{*}(t)$, we have

$$\begin{aligned} \bigl\Vert Q_{1}(t, S)-Q_{1}\bigl(t, S^{*} \bigr) \bigr\Vert =& \bigl\Vert \bigl(b_{1} -\bigl[b_{2}+ \beta (I_{s}+ \beta _{hr}I_{h}+ \beta _{ar}A )+K_{v}\bigr]S+\eta R\bigr) \\ &{}-\bigl(b_{1} -\bigl[b_{2}+ \beta (I_{s}+\beta _{hr}I_{h}+ \beta _{ar}A )+K_{v} \bigr]S+ \eta R\bigr) \bigr\Vert \\ =& \bigl\Vert (b_{2}+\beta I_{s}+ \beta _{hr} I_{h}+\beta _{ar} A+K_{v}) \bigl(S^{*}-S \bigr) \bigr\Vert \\ \leq & \bigl(b_{2}+\beta \Vert I_{s} \Vert + \beta _{hr} \Vert I_{h} \Vert +\beta _{ar} \Vert A \Vert +K_{v}\bigr) \bigl\Vert \bigl(S^{*}-S\bigr) \bigr\Vert \\ \leq &(b_{2}+\beta \psi _{1}+\beta _{hr} \psi _{2})+\beta _{ar} \psi _{3}+K_{v} \bigl\Vert (S-S*) \bigr\Vert \\ \leq &\phi _{1} \bigl\Vert \bigl(S-S^{*}\bigr) \bigr\Vert . \end{aligned}$$

Hence $Q_{1}$ satisfies the Lipschitz condition and $\phi _{1}< 1$. Next we prove that $Q_{2}(t, E)$ satisfies the Lipschitz condition. Now, using $E(t)$, $E^{*}(t)$, we have

$$\begin{aligned} \bigl\Vert Q_{2}(t, E)-Q_{2}\bigl(t, E^{*} \bigr) \bigr\Vert =& \bigl\Vert \bigl(-(b_{2}+\gamma )E+\beta (I_{s}+ \beta _{hr}I_{h}+\beta _{ar}A)S \bigr) \\ &{}-\bigl(-(b_{2}+\gamma )E^{*}+\beta (I_{s}+ \beta _{hr}I_{h}+\beta _{ar}A)S\bigr) \bigr\Vert \\ =& \bigl\Vert (b_{2}+\lambda ) \bigl(E^{*}-E\bigr) \bigr\Vert \\ \leq &(b_{2}+\lambda ) \bigl\Vert \bigl(E^{*}-E\bigr) \bigr\Vert \\ \leq &\phi _{2} \bigl\Vert E-E^{*} \bigr\Vert . \end{aligned}$$

Hence $Q_{2}$ satisfies the Lipschitz condition and $\phi _{2}< 1$. Next we prove that $Q_{3}(t, I_{s})$ satisfies the Lipschitz condition. Using $I_{s}(t)$, $I_{s}^{*}(t)$, we have

$$\begin{aligned} \bigl\Vert Q_{3}(t, I_{s})-Q_{3}\bigl(t, I_{s}^{*}\bigr) \bigr\Vert =& \bigl\Vert \bigl(-(b_{2}+ \tau _{0})I_{s}+ \gamma P_{s} E\bigr)-\bigl(-(b_{2}+ \tau _{0})I_{s}+ \gamma P_{s} E\bigr) \bigr\Vert \\ =& \bigl\Vert (b_{2}+\tau _{0}) \bigl(I_{s}^{*}-I_{s} \bigr) \bigr\Vert \\ \leq &(b_{2}+\tau _{0}) \bigl\Vert \bigl(I_{s}^{*}-I_{s}\bigr) \bigr\Vert \\ \leq &\phi _{3} \bigl\Vert I_{s}-I_{s}^{*} \bigr\Vert . \end{aligned}$$

Hence $Q_{3}$ satisfies the Lipschitz condition and $\phi _{3}< 1$. Next we prove that $Q_{4}(t, I_{h})$ satisfies the Lipschitz condition. Using $I_{h}(t)$, $I_{h}^{*}(t)$, we have

$$\begin{aligned}& \bigl\Vert Q_{4}(t, I_{h})-Q_{4}\bigl(t, I_{h}^{*}\bigr) \bigr\Vert \\& \quad = \bigl\Vert \bigl(-(b_{2}+\alpha + \tau _{0}+K_{T}) I_{h}+\gamma P_{h}E\bigr) \\& \qquad {}-\bigl(-(b_{2}+\alpha + \tau _{0}+K_{T}) I_{h}^{*}+ \gamma P_{h}E\bigr) \bigr\Vert \\& \quad = \|((b_{2}+\alpha + \tau _{0}+K_{T}) \bigl(I_{h}^{*}-I_{h}\bigr)\| \\& \quad \leq ((b_{2}+\alpha + \tau _{0}+K_{T}) \bigl\Vert \bigl(I_{h}^{*}-I_{h}\bigr) \bigr\Vert \\& \quad \leq \phi _{4} \bigl\Vert I_{h}-I_{h}^{*} \bigr\Vert . \end{aligned}$$

Hence $Q_{4}$ satisfies the Lipschitz condition and $\phi _{4}< 1$. Next we prove that $Q_{5}(t, A)$ satisfies the Lipschitz condition. Using $A(t)$, $A^{*}(t)$, we have

$$\begin{aligned}& \bigl\Vert Q_{5}(t, A)-Q_{5}\bigl(t, A^{*} \bigr) \bigr\Vert \\& \quad = \bigl\Vert \bigl(-(b_{2}+ \tau _{0})A + \gamma (1-P_{s}-P_{h})E\bigr) \\& \qquad {}- \bigl(-(b_{2}+ \tau _{0})A^{*} + \gamma (1-P_{s}-P_{h})E\bigr) \bigr\Vert \\& \quad = \bigl\Vert (b_{2}+ \tau _{0}) \bigl(A^{*}-A \bigr) \bigr\Vert \\& \quad \leq \|(b_{2}+ \tau _{0} \bigl\Vert \bigl(A^{*}-A\bigr) \bigr\Vert \\& \quad \leq \phi _{5} \bigl\Vert A-A^{*} \bigr\Vert . \end{aligned}$$

Hence $Q_{5}$ satisfies the Lipschitz condition and $\phi _{5}< 1$. Next we prove that $Q_{6}(t, R)$ satisfies the Lipschitz condition. Using $R(t)$, $R^{*}(t)$, we have

$$\begin{aligned}& \bigl\Vert Q_{6}(t, E_{M})-Q_{6}\bigl(t, R^{*}\bigr) \bigr\Vert \\& \quad = \bigl\Vert (\beta S_{M} I -vE_{M}-\mu _{M}E_{M})-\bigl( \beta S_{M} I -vE_{M}^{*}-\mu _{M}E_{M}^{*} \bigr) \bigr\Vert \\& \quad = \bigl\Vert (b_{2}+\eta ) \bigl(R^{*}-R\bigr) \bigr\Vert \\& \quad \leq (b_{2}+\eta ) \bigl\Vert \bigl(R^{*}-R\bigr) \bigr\Vert \\& \quad \leq \phi _{6} \bigl\Vert R-R^{*} \bigr\Vert . \end{aligned}$$

Hence $Q_{6}$ satisfies the Lipschitz condition and $\phi _{6}< 1$. Ultimately all the functions satisfy Lipschitz conditions and are contractions with $\phi _{i}< 1$ for $i\in N_{1}^{6}$. Hence this completes the proof. □

We rewrite the system of equations (4) in the following form by using the kernels $Q_{i}$, $i\in N_{1}^{6}$ and the initial conditions $S(0)=E(0)=I_{s}(0)=I_{h}(0)=A(0)=R(0)=0 $, we have

6 $$ \textstyle\begin{cases} S(t)=\frac{u_{1} u_{2}}{AB(u_{1})\Gamma u_{1}} \int _{0}^{t} s^{u_{2}-1}(t-s)^{u_{1}-1}Q_{1}(s,S(s))\,ds+ \frac{u_{2}(1-u_{1})t^{u_{2}-1}}{AB(u_{1})}Q_{1}(t,S(t)), \\ E(t)=\frac{u_{1} u_{2}}{AB(u_{1})\Gamma u_{1}} \int _{0}^{t} s^{u_{2}-1}(t-s)^{u_{1}-1}Q_{2}(s,E(s))\,ds+ \frac{u_{2}(1-u_{1})t^{u_{2}-1}}{AB(u_{1})}Q_{2}(t,E(t)), \\ I_{s}(t)=\frac{u_{1} u_{2}}{AB(u_{1})\Gamma u_{1}} \int _{0}^{t} s^{u_{2}-1}(t-s)^{u_{1}-1}Q_{3}(s,I_{s}(s))\,ds+ \frac{u_{2}(1-u_{1})t^{u_{2}-1}}{AB(u_{1})}Q_{3}(t,I_{s}(t)), \\ I_{h}(t)=\frac{u_{1} u_{2}}{AB(u_{1})\Gamma u_{1}} \int _{0}^{t} s^{u_{2}-1}(t-s)^{u_{1}-1}Q_{4}(s,I_{h}(s))\,ds+ \frac{u_{2}(1-u_{1})t^{u_{2}-1}}{AB(u_{1})}Q_{4}(t,I_{h}(t)), \\ A(t)=\frac{u_{1} u_{2}}{AB(u_{1})\Gamma u_{1}} \int _{0}^{t} s^{u_{2}-1}(t-s)^{u_{1}-1}Q_{5}(s,A(s))\,ds+ \frac{u_{2}(1-u_{1})t^{u_{2}-1}}{AB(u_{1})}Q_{5}(t,A(t)), \\ R(t)=\frac{u_{1} u_{2}}{AB(u_{1})\Gamma u_{1}} \int _{0}^{t} s^{u_{2}-1}(t-s)^{u_{1}-1}Q_{6}(s,R(s))\,ds+ \frac{u_{2}(1-u_{1})t^{u_{2}-1}}{AB(u_{1})}Q_{6}(t,R(t)). \end{cases} $$

Now we define the following recursive formulas:

$$\begin{aligned}& S_{n}(t)=\frac{u_{1} u_{2}}{AB(u_{1})\Gamma (u_{1})} \int _{0}^{t} s^{u_{2}-1}(t-s)^{u_{1}-1}Q_{1} \bigl(s,S_{n-1}(s)\bigr)\,ds \\& \hphantom{S_{n}(t)=}{}+ \frac{u_{2}(1-u_{1})t^{u_{2}-1}}{AB(u_{1})}Q_{1} \bigl(t,S_{n-1}(t)\bigr), \\& E_{n}(t)=\frac{u_{1} u_{2}}{AB(u_{1})\Gamma u_{1}} \int _{0}^{t} s^{u_{2}-1}(t-s)^{u_{1}-1}Q_{2} \bigl(s,E_{n-1}(s)\bigr)\,ds \\& \hphantom{E_{n}(t)=}{}+ \frac{u_{2}(1-u_{1})t^{u_{2}-1}}{AB(u_{1})}Q_{2} \bigl(t,E_{n-1}(t)\bigr), \\& I_{s_{n}}(t)=\frac{u_{1} u_{2}}{AB(u_{1})\Gamma u_{1}} \int _{0}^{t} s^{u_{2}-1}(t-s)^{u_{1}-1}Q_{3} \bigl(s,I_{s_{n-1}}(s)\bigr)\,ds \\& \hphantom{I_{s_{n}}(t)=}{}+ \frac{u_{2}(1-u_{1})t^{u_{2}-1}}{AB(u_{1})}Q_{3} \bigl(t,I_{s_{n-1}}(t)\bigr), \\& I_{h_{n}}(t)=\frac{u_{1} u_{2}}{AB(u_{1})\Gamma u_{1}} \int _{0}^{t} s^{u_{2}-1}(t-s)^{u_{1}-1}Q_{4} \bigl(s,I_{h_{n-1}}(s)\bigr)\,ds \\& \hphantom{I_{h_{n}}(t)=}{}+ \frac{u_{2}(1-u_{1})t^{u_{2}-1}}{AB(u_{1})}Q_{4} \bigl(t,I_{h_{n-1}}(t)\bigr), \\& A_{n}(t)=\frac{u_{1} u_{2}}{AB(u_{1})\Gamma u_{1}} \int _{0}^{t} s^{u_{2}-1}(t-s)^{u_{1}-1}Q_{5} \bigl(s,A_{n-1}(s)\bigr)\,ds \\& \hphantom{A_{n}(t)=}{}+ \frac{u_{2}(1-u_{1})t^{u_{2}-1}}{AB(u_{1})}Q_{5} \bigl(t,A_{n-1}(t)\bigr), \\& R_{n}(t)=\frac{u_{1} u_{2}}{AB(u_{1})\Gamma u_{1}} \int _{0}^{t} s^{u_{2}-1}(t-s)^{u_{1}-1}Q_{6} \bigl(s,R_{n-1}(s)\bigr)\,ds \\& \hphantom{R_{n}(t)=}{}+ \frac{u_{2}(1-u_{1})t^{u_{2}-1}}{AB(u_{1})}Q_{6} \bigl(t,R_{n-1}(t)\bigr). \end{aligned}$$

Now we consider the following differences:

$$\begin{aligned}& D S_{n+1}(t) = S_{n+1}-S_{n} \\& \hphantom{D S_{n+1}(t)} = \frac{u_{1} u_{2}}{AB(u_{1})\Gamma u_{1}} \int _{0}^{t} s^{u_{2}-1}(t-s)^{u_{1}-1}Q_{1} \bigl(s,S_{n}(s)\bigr)\,ds+ \frac{u_{2}(1-u_{1})t^{u_{2}-1}}{AB(u_{1})}Q_{1} \bigl(t,S_{n}(t)\bigr) \\& \hphantom{D S_{n+1}(t)=} {}- \biggl(\frac{u_{1} u_{2}}{AB(u_{1})\Gamma u_{1}} \int _{0}^{t} s^{u_{2}-1}(t-s)^{u_{1}-1}Q_{1} \bigl(s,S_{n-1}(s)\bigr)\,ds \\& \hphantom{D S_{n+1}(t)=} {} + \frac{u_{2}(1-u_{1})t^{u_{2}-1}}{AB(u_{1})}Q_{1}\bigl(t,S_{n-1}(t)\bigr) \biggr) \\& \hphantom{D S_{n+1}(t)} = \frac{u_{1} u_{2}}{AB(u_{1})\Gamma u_{1}} \int _{0}^{t} s^{u_{2}-1}(t-s)^{u_{1}-1} \bigl(Q_{1}\bigl(s,S_{n}(s)\bigr)-Q_{1} \bigl(s,S_{n-1}(s)\bigr) \bigr)\,ds \\& \hphantom{D S_{n+1}(t)=} {} + \frac{u_{2}(1-u_{1})t^{u_{2}-1}}{AB(u_{1})} \bigl(Q_{1}\bigl(t,S_{n}(t) \bigr)-Q_{1}\bigl(t,S_{n-1}(t)\bigr) \bigr), \\& D E_{n+1}(t) = E_{n+1}-E_{n} \\& \hphantom{D E_{n+1}(t)} = \frac{u_{1} u_{2}}{AB(u_{1})\Gamma u_{1}} \int _{0}^{t} s^{u_{2}-1}(t-s)^{u_{1}-1}Q_{2} \bigl(s,E_{n}(s)\bigr)\,ds+ \frac{u_{2}(1-u_{1})t^{u_{2}-1}}{AB(u_{1})}Q_{2} \bigl(t,E_{n}(t)\bigr) \\& \hphantom{D E_{n+1}(t)=} {}- \biggl(\frac{u_{1} u_{2}}{AB(u_{1})\Gamma u_{1}} \int _{0}^{t} s^{u_{2}-1}(t-s)^{u_{1}-1}Q_{2} \bigl(s,E_{n-1}(s)\bigr)\,ds \\& \hphantom{D E_{n+1}(t)=} {}+ \frac{u_{2}(1-u_{1})t^{u_{2}-1}}{AB(u_{1})}Q_{2} \bigl(t,E_{n-1}(t)\bigr) \biggr) \\& \hphantom{D E_{n+1}(t)} = \frac{u_{1} u_{2}}{AB(u_{1})\Gamma u_{1}} \int _{0}^{t} s^{u_{2}-1}(t-s)^{u_{1}-1} \bigl(Q_{2}\bigl(s,E_{n}(s)\bigr)-Q_{1} \bigl(s,E_{n-1}(s)\bigr) \bigr)\,ds \\& \hphantom{D E_{n+1}(t)=}{} + \frac{u_{2}(1-u_{1})t^{u_{2}-1}}{AB(u_{1})} \bigl(Q_{2}\bigl(t,E_{n}(t) \bigr)-Q_{2}\bigl(t,E_{n-1}(t)\bigr) \bigr), \\& D I_{s_{n+1}}(t) = I_{s_{n+1}}-I_{H_{n}} \\& \hphantom{D I_{s_{n+1}}(t)} = \frac{u_{1} u_{2}}{AB(u_{1})\Gamma u_{1}} \int _{0}^{t} s^{u_{2}-1}(t-s)^{u_{1}-1}Q_{3} \bigl(s,I_{s_{n}}(s)\bigr)\,ds+ \frac{u_{2}(1-u_{1})t^{u_{2}-1}}{AB(u_{1})}Q_{3} \bigl(t,I_{s_{n}}(t)\bigr) \\& \hphantom{D I_{s_{n+1}}(t)=} {}- \biggl(\frac{u_{1} u_{2}}{AB(u_{1})\Gamma u_{1}} \int _{0}^{t} s^{u_{2}-1}(t-s)^{u_{1}-1}Q_{3} \bigl(s,I_{s_{n-1}}(s)\bigr)\,ds \\& \hphantom{D I_{s_{n+1}}(t)=} {}+ \frac{u_{2}(1-u_{1})t^{u_{2}-1}}{AB(u_{1})}Q_{3} \bigl(t,I_{s_{n-1}}(t)\bigr) \biggr) \\& \hphantom{D I_{s_{n+1}}(t)} = \frac{u_{1} u_{2}}{AB(u_{1})\Gamma u_{1}} \int _{0}^{t} s^{u_{2}-1}(t-s)^{u_{1}-1} \bigl(Q_{3}\bigl(s,I_{s_{n}}(s)\bigr)-Q_{3} \bigl(s,I_{s_{n-1}}(s)\bigr) \bigr)\,ds \\& \hphantom{D I_{s_{n+1}}(t)=}{} + \frac{u_{2}(1-u_{1})t^{u_{2}-1}}{AB(u_{1})} \bigl(Q_{3}\bigl(t,I_{s_{n}}(t) \bigr)-Q_{3}\bigl(t,I_{s_{n-1}}(t)\bigr) \bigr), \\& D I_{h_{n+1}}(t) = I_{h_{n+1}}-I_{h_{n}} \\& \hphantom{D I_{h_{n+1}}(t)} = \frac{u_{1} u_{2}}{AB(u_{1})\Gamma u_{1}} \int _{0}^{t} s^{u_{2}-1}(t-s)^{u_{1}-1}Q_{4} \bigl(s,I_{h_{n}}(s)\bigr)\,ds+ \frac{u_{2}(1-u_{1})t^{u_{2}-1}}{AB(u_{1})}Q_{4} \bigl(t,I_{h_{n}}(t)\bigr) \\& \hphantom{D I_{h_{n+1}}(t)=} {}- \biggl(\frac{u_{1} u_{2}}{AB(u_{1})\Gamma u_{1}} \int _{0}^{t} s^{u_{2}-1}(t-s)^{u_{1}-1}Q_{4} \bigl(s,I_{h_{n-1}}(s)\bigr)\,ds \\& \hphantom{D I_{h_{n+1}}(t)=} {}+ \frac{u_{2}(1-u_{1})t^{u_{2}-1}}{AB(u_{1})}Q_{4} \bigl(t,I_{h_{n-1}}(t)\bigr) \biggr) \\& \hphantom{D I_{h_{n+1}}(t)}= \frac{u_{1} u_{2}}{AB(u_{1})\Gamma u_{1}} \int _{0}^{t} s^{u_{2}-1}(t-s)^{u_{1}-1} \bigl(Q_{4}\bigl(s,I_{h_{n}}(s)\bigr)-Q_{4} \bigl(s,I_{h_{n-1}}(s)\bigr) \bigr)\,ds \\& \hphantom{D I_{h_{n+1}}(t)=}{}+ \frac{u_{2}(1-u_{1})t^{u_{2}-1}}{AB(u_{1})} \bigl(Q_{4}\bigl(t,I_{h_{n}}(t) \bigr)-Q_{4}\bigl(t,I_{h_{n-1}}(t)\bigr) \bigr), \\& D A_{n+1}(t) = A_{n+1}-A_{n} \\& \hphantom{D A_{n+1}(t)}= \frac{u_{1} u_{2}}{AB(u_{1})\Gamma u_{1}} \int _{0}^{t} s^{u_{2}-1}(t-s)^{u_{1}-1}Q_{5} \bigl(s,A_{n}(s)\bigr)\,ds+ \frac{u_{2}(1-u_{1})t^{u_{2}-1}}{AB(u_{1})}Q_{5} \bigl(t,A_{n}(t)\bigr) \\& \hphantom{D A_{n+1}(t)=} {}- \biggl(\frac{u_{1} u_{2}}{AB(u_{1})\Gamma u_{1}} \int _{0}^{t} s^{u_{2}-1}(t-s)^{u_{1}-1}Q_{5} \bigl(s,A_{n-1}(s)\bigr)\,ds \\& \hphantom{D A_{n+1}(t)=} {}+ \frac{u_{2}(1-u_{1})t^{u_{2}-1}}{AB(u_{1})}Q_{5} \bigl(t,A_{n-1}(t)\bigr) \biggr) \\& \hphantom{D A_{n+1}(t)} = \frac{u_{1} u_{2}}{AB(u_{1})\Gamma u_{1}} \int _{0}^{t} s^{u_{2}-1}(t-s)^{u_{1}-1} \bigl(Q_{5}\bigl(s,A_{n}(s)\bigr)-Q_{5} \bigl(s,A_{n-1}(s)\bigr) \bigr)\,ds \\& \hphantom{D A_{n+1}(t)=}{} + \frac{u_{2}(1-u_{1})t^{u_{2}-1}}{AB(u_{1})} \bigl(Q_{5}\bigl(t,A_{n}(t) \bigr)-Q_{5}\bigl(t,A_{n-1}(t)\bigr) \bigr), \\& D R_{n+1}(t) = R_{n+1}-R_{n} \\& \hphantom{D R_{n+1}(t)} = \frac{u_{1} u_{2}}{AB(u_{1})\Gamma u_{1}} \int _{0}^{t} s^{u_{2}-1}(t-s)^{u_{1}-1}Q_{6} \bigl(s,R_{n}(s)\bigr)\,ds+ \frac{u_{2}(1-u_{1})t^{u_{2}-1}}{AB(u_{1})}Q_{6} \bigl(t,R_{n}(t)\bigr) \\& \hphantom{D R_{n+1}(t)=} {}- \biggl(\frac{u_{1} u_{2}}{AB(u_{1})\Gamma u_{1}} \int _{0}^{t} s^{u_{2}-1}(t-s)^{u_{1}-1}Q_{6} \bigl(s,R_{n-1}(s)\bigr)\,ds \\& \hphantom{D R_{n+1}(t)=}{}+ \frac{u_{2}(1-u_{1})t^{u_{2}-1}}{AB(u_{1})}Q_{6} \bigl(t,R_{n-1}(t)\bigr) \biggr) \\& \hphantom{D R_{n+1}(t)} = \frac{u_{1} u_{2}}{AB(u_{1})\Gamma u_{1}} \int _{0}^{t} s^{u_{2}-1}(t-s)^{u_{1}-1} \bigl(Q_{6}\bigl(s,R_{n}(s)\bigr)-Q_{6} \bigl(s,R_{n-1}(s)\bigr) \bigr)\,ds \\& \hphantom{D R_{n+1}(t)=}{} + \frac{u_{2}(1-u_{1})t^{u_{2}-1}}{AB(u_{1})} \bigl(Q_{6}\bigl(t,R_{n}(t) \bigr)-Q_{6}\bigl(t,R_{n-1}(t)\bigr) \bigr). \end{aligned}$$

Taking norm of the above differences, we have

$$\begin{aligned}& \bigl\Vert D S_{n+1}(t) \bigr\Vert \\& \quad = \Vert S_{n+1}-S_{n} \Vert \\& \quad = \biggl\Vert \frac{u_{1} u_{2}}{AB(u_{1})\Gamma u_{1}} \int _{0}^{t} s^{u_{2}-1}(t-s)^{u_{1}-1}Q_{1} \bigl(s,S_{n}(s)\bigr)\,ds+ \frac{u_{2}(1-u_{1})t^{u_{2}-1}}{AB(u_{1})}Q_{1} \bigl(t,S_{n}(t)\bigr) \\& \qquad {}- \biggl(\frac{u_{1} u_{2}}{AB(u_{1})\Gamma u_{1}} \int _{0}^{t} s^{u_{2}-1}(t-s)^{u_{1}-1}Q_{1} \bigl(s,S_{n-1}(s)\bigr)\,ds \\& \qquad {}+ \frac{u_{2}(1-u_{1})t^{u_{2}-1}}{AB(u_{1})}Q_{1} \bigl(t,S_{n-1}(t)\bigr) \biggr) \biggr\Vert \\& \quad = \frac{u_{1} u_{2}}{AB(u_{1})\Gamma u_{1}} \int _{0}^{t} s^{u_{2}-1}(t-s)^{u_{1}-1} \bigl\Vert Q_{1}\bigl(s,S_{n}(s)\bigr)-Q_{1} \bigl(s,S_{n-1}(s)\bigr) \bigr\Vert \,ds \\& \qquad {} + \frac{u_{2}(1-u_{1})t^{u_{2}-1}}{AB(u_{1})} \bigl\Vert Q_{1}\bigl(t,S_{n}(t) \bigr)-Q_{1}\bigl(t,S_{n-1}(t)\bigr) \bigr\Vert , \\& \bigl\Vert D E_{n+1}(t) \bigr\Vert \\& \quad = \Vert E_{n+1}-E_{n} \Vert \\& \quad = \biggl\Vert \frac{u_{1} u_{2}}{AB(u_{1})\Gamma u_{1}} \int _{0}^{t} s^{u_{2}-1}(t-s)^{u_{1}-1}Q_{2} \bigl(s,E_{n}(s)\bigr)\,ds+ \frac{u_{2}(1-u_{1})t^{u_{2}-1}}{AB(u_{1})}Q_{2} \bigl(t,E_{n}(t)\bigr) \\& \qquad {}- \biggl(\frac{u_{1} u_{2}}{AB(u_{1})\Gamma u_{1}} \int _{0}^{t} s^{u_{2}-1}(t-s)^{u_{1}-1}Q_{2} \bigl(s,E_{n-1}(s)\bigr)\,ds \\& \qquad {}+ \frac{u_{2}(1-u_{1})t^{u_{2}-1}}{AB(u_{1})}Q_{2} \bigl(t,E_{n-1}(t)\bigr) \biggr) \biggr\Vert \\& \quad = \frac{u_{1} u_{2}}{AB(u_{1})\Gamma u_{1}} \int _{0}^{t} s^{u_{2}-1}(t-s)^{u_{1}-1} \bigl\Vert \bigl(Q_{2}\bigl(s,E_{n}(s) \bigr)-Q_{1}\bigl(s,E_{n-1}(s)\bigr)\bigr) \bigr\Vert \,ds \\& \qquad {} + \frac{u_{2}(1-u_{1})t^{u_{2}-1}}{AB(u_{1})}\|(Q_{2}\bigl(t,E_{n}(t) \bigr)-Q_{2}\bigl(t,E_{n-1}(t)\bigr) \|, \\& \bigl\Vert D I_{s_{n+1}}(t) \bigr\Vert \\& \quad = \Vert I_{s_{n+1}}-I_{s_{n}} \Vert \\& \quad = \biggl\Vert \frac{u_{1} u_{2}}{AB(u_{1})\Gamma u_{1}} \int _{0}^{t} s^{u_{2}-1}(t-s)^{u_{1}-1}Q_{3} \bigl(s,I_{s_{n}}(s)\bigr)\,ds+ \frac{u_{2}(1-u_{1})t^{u_{2}-1}}{AB(u_{1})}Q_{3} \bigl(t,I_{s_{n}}(t)\bigr) \\& \qquad {} {}- \biggl(\frac{u_{1} u_{2}}{AB(u_{1})\Gamma u_{1}} \int _{0}^{t} s^{u_{2}-1}(t-s)^{u_{1}-1}Q_{3} \bigl(s,I_{s_{n-1}}(s)\bigr)\,ds \\& \qquad {} +\frac{u_{2}(1-u_{1})t^{u_{2}-1}}{AB(u_{1})}Q_{3} \bigl(t,I_{s_{n-1}}(t)\bigr) \biggr) \biggr\Vert \\& \quad = \frac{u_{1} u_{2}}{AB(u_{1})\Gamma u_{1}} \int _{0}^{t} s^{u_{2}-1}(t-s)^{u_{1}-1} \| \bigl(Q_{3}\bigl(s,I_{s_{n}}(s)\bigr)-Q_{3} \bigl(s,I_{s_{n-1}}(s)\bigr)\|\bigr)\,ds \\& \qquad {} + \frac{u_{2}(1-u_{1})t^{u_{2}-1}}{AB(u_{1})} \bigl\Vert Q_{3}\bigl(t,I_{s_{n}}(t) \bigr)-Q_{3}\bigl(t,I_{s_{n-1}}(t)\bigr) \bigr\Vert , \\& \bigl\Vert D I_{h_{n+1}}(t) \bigr\Vert \\& \quad = \Vert I_{h_{n+1}}-I_{h_{n}} \Vert \\& \quad = \biggl\Vert \frac{u_{1} u_{2}}{AB(u_{1})\Gamma u_{1}} \int _{0}^{t} s^{u_{2}-1}(t-s)^{u_{1}-1}Q_{4} \bigl(s,I_{h_{n}}(s)\bigr)\,ds+ \frac{u_{2}(1-u_{1})t^{u_{2}-1}}{AB(u_{1})}Q_{4} \bigl(t,I_{h_{n}}(t)\bigr) \\& \qquad {}- \biggl(\frac{u_{1} u_{2}}{AB(u_{1})\Gamma u_{1}} \int _{0}^{t} s^{u_{2}-1}(t-s)^{u_{1}-1}Q_{4} \bigl(s,I_{h_{n-1}}(s)\bigr)\,ds \\& \qquad {}+ \frac{u_{2}(1-u_{1})t^{u_{2}-1}}{AB(u_{1})}Q_{4} \bigl(t,I_{h_{n-1}}(t)\bigr) \biggr) \biggr\Vert \\& \quad = \frac{u_{1} u_{2}}{AB(u_{1})\Gamma u_{1}} \int _{0}^{t} s^{u_{2}-1}(t-s)^{u_{1}-1} \bigl\Vert Q_{4}\bigl(s,I_{h_{n}}(s)\bigr)-Q_{4} \bigl(s,I_{h_{n-1}}(s)\bigr) \bigr\Vert \,ds \\& \qquad {} + \frac{u_{2}(1-u_{1})t^{u_{2}-1}}{AB(u_{1})} \bigl\Vert Q_{4}\bigl(t,I_{h_{n}}(t) \bigr)-Q_{4}\bigl(t,I_{h_{n-1}}(t)\bigr) \bigr\Vert , \\& \bigl\Vert D A_{n+1}(t) \bigr\Vert \\& \quad = \Vert A_{n+1}-A_{n} \Vert \\& \quad = \biggl\Vert \frac{u_{1} u_{2}}{AB(u_{1})\Gamma u_{1}} \int _{0}^{t} s^{u_{2}-1}(t-s)^{u_{1}-1}Q_{5} \bigl(s,A_{n}(s)\bigr)\,ds+ \frac{u_{2}(1-u_{1})t^{u_{2}-1}}{AB(u_{1})}Q_{5} \bigl(t,A_{n}(t)\bigr) \\& \qquad {}- \biggl(\frac{u_{1} u_{2}}{AB(u_{1})\Gamma u_{1}} \int _{0}^{t} s^{u_{2}-1}(t-s)^{u_{1}-1}Q_{5} \bigl(s,A_{n-1}(s)\bigr)\,ds \\& \qquad {}+ \frac{u_{2}(1-u_{1})t^{u_{2}-1}}{AB(u_{1})}Q_{5} \bigl(t,A_{n-1}(t)\bigr) \biggr) \biggr\Vert \\& \quad = \frac{u_{1} u_{2}}{AB(u_{1})\Gamma u_{1}} \int _{0}^{t} s^{u_{2}-1}(t-s)^{u_{1}-1} \bigl\Vert Q_{5}\bigl(s,A_{n}(s)\bigr)-Q_{5} \bigl(s,A_{n-1}(s)\bigr) \bigr\Vert \,ds \\& \qquad {} + \frac{u_{2}(1-u_{1})t^{u_{2}-1}}{AB(u_{1})} \bigl\Vert Q_{5}\bigl(t,A_{n}(t) \bigr)-Q_{5}\bigl(t,A_{n-1}(t)\bigr) \bigr\Vert , \\& \bigl\Vert D R_{n+1}(t) \bigr\Vert \\& \quad = \Vert R_{n+1}-R_{n} \Vert \\& \quad = \biggl\Vert \frac{u_{1} u_{2}}{AB(u_{1})\Gamma u_{1}} \int _{0}^{t} s^{u_{2}-1}(t-s)^{u_{1}-1}Q_{6} \bigl(s,R_{n}(s)\bigr)\,ds+ \frac{u_{2}(1-u_{1})t^{u_{2}-1}}{AB(u_{1})}Q_{6} \bigl(t,R_{n}(t)\bigr) \\& \qquad {}- \biggl(\frac{u_{1} u_{2}}{AB(u_{1})\Gamma u_{1}} \int _{0}^{t} s^{u_{2}-1}(t-s)^{u_{1}-1}Q_{6} \bigl(s,R_{n-1}(s)\bigr)\,ds \\& \qquad {}+ \frac{u_{2}(1-u_{1})t^{u_{2}-1}}{AB(u_{1})}Q_{6} \bigl(t,R_{n-1}(t)\bigr) \biggr) \biggr\Vert \\& \quad = \frac{u_{1} u_{2}}{AB(u_{1})\Gamma u_{1}} \int _{0}^{t} s^{u_{2}-1}(t-s)^{u_{1}-1} \bigl\Vert Q_{6}\bigl(s,R_{n}(s)\bigr)-Q_{6} \bigl(s,R_{n-1}(s)\bigr) \bigr\Vert \,ds \\& \qquad {} + \frac{u_{2}(1-u_{1})t^{u_{2}-1}}{AB(u_{1})} \bigl\Vert Q_{6}\bigl(t,R_{n}(t) \bigr)-Q_{6}\bigl(t,R_{n-1}(t)\bigr) \bigr\Vert . \end{aligned}$$

### Theorem 2.2

*The fractal fractional of diffusion model SE*(*Is*)(*Ih*)*AR epidemic has a solution if the following holds true*:

$$\sigma =\max \{\phi _{1},\phi _{2},\ldots, \phi _{6}\}< 1. $$

### Proof

Let us define the following functions:

7 $$ \textstyle\begin{cases} G_{1}n(t)=S_{n+1}(t)-S(t), \\ G_{2}n(t)=E_{n+1}(t)-E(t), \\ G_{3}n(t)=I_{s_{n+1}}(t)-I_{s}(t), \\ G_{4}n(t)=I_{h_{n+1}}(t)-I_{h}(t), \\ G_{5}n(t)=A_{n+1}(t)-A(t), \\ G_{6}n(t)=R_{n+1}(t)-R(t). \end{cases} $$

Taking norm of the above system, we have

$$\begin{aligned}& \bigl\Vert G_{1}n(t) \bigr\Vert \\& \quad = \bigl\Vert S_{n+1}(t)-S(t) \bigr\Vert \\& \quad = \biggl\Vert \frac{u_{1} u_{2}}{AB(u_{1})\Gamma u_{1}} \int _{0}^{t} s^{u_{2}-1}(t-s)^{u_{1}-1}Q_{1} \bigl(s,S_{n}(s)\bigr)\,ds+ \frac{u_{2}(1-u_{1})t^{u_{2}-1}}{AB(u_{1})}Q_{1} \bigl(t,S_{n}(t)\bigr) \\& \qquad {}- \biggl(\frac{u_{1} u_{2}}{AB(u_{1})\Gamma u_{1}} \int _{0}^{t} s^{u_{2}-1}(t-s)^{u_{1}-1}Q_{1}(s,S) (s)\biggr)\,ds+ \frac{u_{2}(1-u_{1})t^{u_{2}-1}}{AB(u_{1})}Q_{1}(t,S) (t)) ) \biggr\Vert \\& \quad = \frac{u_{1} u_{2}}{AB(u_{1})\Gamma u_{1}} \int _{0}^{t} s^{u_{2}-1}(t-s)^{u_{1}-1} \bigl\Vert Q_{1}\bigl(s,S_{n}(s)\bigr)-Q_{1}(s,S) (s) \bigr\Vert \,ds \\& \qquad {} + \frac{u_{2}(1-u_{1})t^{u_{2}-1}}{AB(u_{1})} \bigl\Vert Q_{1}\bigl(t,S_{n}(t) \bigr)-Q_{1}(t,S) (t) \bigr\Vert \\& \quad \leq \biggl(\frac{u_{1} u_{2}}{AB(u_{1})\Gamma u_{1}} \int _{0}^{t} s^{u_{2}-1}(t-s)^{u_{1}-1}+ \frac{u_{2}(1-u_{1})t^{u_{2}-1}}{ AB(u_{1})} \biggr)\phi _{1} \Vert S_{n}-S \Vert \\& \quad \leq \biggl( \frac{u_{1} u_{2}\Gamma u_{2}}{AB(u_{1}\Gamma (u_{1}+u_{2}))}+ \frac{u_{2}(1-u_{1})}{AB(u_{1})} \biggr)\phi _{1} \Vert S_{n}-S \Vert \\& \quad \leq \biggl( \frac{u_{1} u_{2}\Gamma u_{2}}{AB(u_{1}\Gamma (u_{1}+u_{2}))}+ \frac{u_{2}(1-u_{1})}{AB(u_{1})} \biggr)^{n} \sigma ^{n} \Vert S_{1}-S \Vert , \end{aligned}$$

where $\sigma < 1$ and as $n \rightarrow \infty $ so $S_{n}\rightarrow S$, and using the formula $B(u, v)=(b-a)^{-u+v+1}\int _{a}^{b}(s-a)^{u-1}(b-s)^{v-1}\,ds$ and as $t\in [0, 1]$ so $t^{-1-u_{1}+u_{2}}\leq 1$ and $t^{u_{2}}\leq 1$,

$$\begin{aligned} \bigl\Vert G_{2}n(t) \bigr\Vert =& \bigl\Vert E_{n+1}(t)-E(t) \bigr\Vert \\ =& \biggl\Vert \frac{u_{1} u_{2}}{AB(u_{1})\Gamma u_{1}} \int _{0}^{t} s^{u_{2}-1}(t-s)^{u_{1}-1}Q_{2} \bigl(s,E_{n}(s)\bigr)\,ds \\ &{}+ \frac{u_{2}(1-u_{1})t^{u_{2}-1}}{AB(u_{1})}Q_{2} \bigl(t,E_{n}(t)\bigr) \\ &{}- \biggl(\frac{u_{1} u_{2}}{AB(u_{1})\Gamma u_{1}} \int _{0}^{t} s^{u_{2}-1}(t-s)^{u_{1}-1}Q_{2}(s,E) (s)\biggr)\,ds \\ &{}+ \frac{u_{2}(1-u_{1})t^{u_{2}-1}}{AB(u_{1})}Q_{2}\bigl(t,E(t)\bigr) ) \biggr\Vert \\ =&\frac{u_{1} u_{2}}{AB(u_{1})\Gamma u_{1}} \int _{0}^{t} s^{u_{2}-1}(t-s)^{u_{1}-1} \big\| Q_{2}\bigl(s,E_{n}(s)\bigr)-Q_{2}(s,E) (s)) \big\Vert \,ds \\ &{}+\frac{u_{2}(1-u_{1})t^{u_{2}-1}}{AB(u_{1})} \big\Vert Q_{2}\bigl(t,E_{n}(t) \bigr)-Q_{2}(t,E) (t)) \big\| \\ \leq & \biggl(\frac{u_{1} u_{2}}{AB(u_{1})\Gamma u_{1}} \int _{0}^{t} s^{u_{2}-1}(t-s)^{u_{1}-1}+ \frac{u_{2}(1-u_{1})t^{u_{2}-1}}{AB(u_{1})} \biggr)\phi _{2} \Vert E_{n}-E \Vert \\ \leq & \biggl( \frac{u_{1} u_{2}\Gamma u_{2}}{AB(u_{1}\Gamma (u_{1}+u_{2}))}+ \frac{u_{2}(1-u_{1})}{AB(u_{1})} \biggr)\phi _{2} \Vert E_{n}-E \Vert \\ \leq & \biggl( \frac{u_{1} u_{2}\Gamma u_{2}}{AB(u_{1}\Gamma (u_{1}+u_{2}))}+ \frac{u_{2}(1-u_{1})}{AB(u_{1})} \biggr)^{n} \sigma ^{n} \Vert E_{1}-E \Vert , \end{aligned}$$

where $\sigma < 1$ and as $n \rightarrow \infty $ so $E_{H_{n}}\rightarrow E$.

$$\begin{aligned}& \bigl\Vert G_{3}n(t) \bigr\Vert \\& \quad = \bigl\Vert I_{s_{n+1}}(t)-I_{s}(t) \bigr\Vert \\& \quad = \biggl\Vert \frac{u_{1} u_{2}}{AB(u_{1})\Gamma u_{1}} \int _{0}^{t} s^{u_{2}-1}(t-s)^{u_{1}-1}Q_{3} \bigl(s,I_{s_{n}}(s)\bigr)\,ds \\& \qquad {}+ \frac{u_{2}(1-u_{1})t^{u_{2}-1}}{AB(u_{1})}Q_{3} \bigl(t,I_{s_{n}}(t)\bigr) \\& \qquad {}- \biggl(\frac{u_{1} u_{2}}{AB(u_{1})\Gamma u_{1}} \int _{0}^{t} s^{u_{2}-1}(t-s)^{u_{1}-1}Q_{3} \bigl(s,I_{s}(s)\bigr)\,ds+ \frac{u_{2}(1-u_{1})t^{u_{2}-1}}{AB(u_{1})}Q_{3} \bigl(t,I_{s}(t)\bigr) \biggr) \biggr\Vert \\& \quad = \frac{u_{1} u_{2}}{AB(u_{1})\Gamma u_{1}} \int _{0}^{t} s^{u_{2}-1}(t-s)^{u_{1}-1} \bigl\Vert Q_{3}\bigl(s,I_{s_{n}}(s)\bigr)-Q_{3} \bigl(s,I_{s}(s)\bigr) \bigr\Vert \,ds \\& \qquad {} + \frac{u_{2}(1-u_{1})t^{u_{2}-1}}{AB(u_{1})} \bigl\Vert Q_{3}\bigl(t,I_{s_{n}}(t) \bigr)-Q_{3}\bigl(t,I_{s}(t)\bigr) \bigr\Vert \\& \quad \leq \biggl(\frac{u_{1} u_{2}}{AB(u_{1})\Gamma u_{1}} \int _{0}^{t} s^{u_{2}-1}(t-s)^{u_{1}-1}+ \frac{u_{2}(1-u_{1})t^{u_{2}-1}}{AB(u_{1})} \biggr)\phi _{3} \Vert I_{s_{n}}-I_{s} \Vert \\& \quad \leq \biggl( \frac{u_{1} u_{2}\Gamma u_{2}}{AB(u_{1}\Gamma (u_{1}+u_{2}))}+ \frac{u_{2}(1-u_{1})}{AB(u_{1})} \biggr)\phi _{3} \Vert I_{s_{n}}-I_{s} \Vert \\& \quad \leq \biggl( \frac{u_{1} u_{2}\Gamma u_{2}}{AB(u_{1}\Gamma (u_{1}+u_{2}))}+ \frac{u_{2}(1-u_{1})}{AB(u_{1})} \biggr)^{n} \sigma ^{n} \Vert I_{s_{1}}-I_{s} \Vert , \end{aligned}$$

where $\sigma < 1$ and as $n \rightarrow \infty $ so $I_{s_{n}}\rightarrow I_{s}$.

$$\begin{aligned}& \bigl\Vert G_{4}n(t) \bigr\Vert \\& \quad = \bigl\Vert I_{h_{n+1}}(t)-I_{h}(t) \bigr\Vert \\& \quad = \biggl\Vert \frac{u_{1} u_{2}}{AB(u_{1})\Gamma u_{1}} \int _{0}^{t} s^{u_{2}-1}(t-s)^{u_{1}-1}Q_{4} \bigl(s,I_{h_{n}}(s)\bigr)\,ds+ \frac{u_{2}(1-u_{1})t^{u_{2}-1}}{AB(u_{1})}Q_{4} \bigl(t,I_{h_{n}}(t)\bigr) \\& \qquad {}- \biggl(\frac{u_{1} u_{2}}{AB(u_{1})\Gamma u_{1}} \int _{0}^{t} s^{u_{2}-1}(t-s)^{u_{1}-1}Q_{4} \bigl(s,I_{h}(s)\bigr)\,ds+ \frac{u_{2}(1-u_{1})t^{u_{2}-1}}{AB(u_{1})}Q_{4} \bigl(t,I_{h}(t)\bigr) \biggr) \biggr\Vert \\& \quad = \frac{u_{1} u_{2}}{AB(u_{1})\Gamma u_{1}} \int _{0}^{t} s^{u_{2}-1}(t-s)^{u_{1}-1} \bigl\Vert Q_{4}\bigl(s,I_{h_{n}}(s)\bigr)-Q_{4} \bigl(s,I_{h}(s)\bigr) \bigr\Vert \,ds \\& \qquad {}+ \frac{u_{2}(1-u_{1})t^{u_{2}-1}}{AB(u_{1})} \bigl\Vert Q_{4}\bigl(t,I_{h_{n}}(t) \bigr)-Q_{4}\bigl(t,I_{h}(t)\bigr) \bigr\Vert \\& \quad \leq \biggl(\frac{u_{1} u_{2}}{AB(u_{1})\Gamma u_{1}} \int _{0}^{t} s^{u_{2}-1}(t-s)^{u_{1}-1}+ \frac{u_{2}(1-u_{1})t^{u_{2}-1}}{AB(u_{1})} \biggr)\phi _{4} \Vert I_{h_{n}}-I_{h} \Vert \\& \quad \leq \biggl( \frac{u_{1} u_{2}\Gamma u_{2}}{AB(u_{1}\Gamma (u_{1}+u_{2}))}+ \frac{u_{2}(1-u_{1})}{AB(u_{1})} \biggr)\phi _{4} \Vert I_{h_{n}}-I_{h} \Vert \\& \quad \leq \biggl( \frac{u_{1} u_{2}\Gamma u_{2}}{AB(u_{1}\Gamma (u_{1}+u_{2}))}+ \frac{u_{2}(1-u_{1})}{AB(u_{1})} \biggr)^{n} \sigma ^{n} \Vert I_{h_{1}}-I_{h} \Vert , \end{aligned}$$

where $\sigma < 1$ and as $n \rightarrow \infty $ so $I_{h_{n}}\rightarrow I_{h}$.

$$\begin{aligned}& \bigl\Vert G_{5}n(t) \bigr\Vert \\& \quad = \bigl\Vert A_{n+1}(t)-A(t) \bigr\Vert \\& \quad = \biggl\Vert \frac{u_{1} u_{2}}{AB(u_{1})\Gamma u_{1}} \int _{0}^{t} s^{u_{2}-1}(t-s)^{u_{1}-1}Q_{5} \bigl(s,A_{n}(s)\bigr)\,ds+ \frac{u_{2}(1-u_{1})t^{u_{2}-1}}{AB(u_{1})}Q_{5} \bigl(t,A_{n}(t)\bigr) \\& \qquad {}- \biggl(\frac{u_{1} u_{2}}{AB(u_{1})\Gamma u_{1}} \int _{0}^{t} s^{u_{2}-1}(t-s)^{u_{1}-1}Q_{5}(s,A) (s)\biggr)\,ds+ \frac{u_{2}(1-u_{1})t^{u_{2}-1}}{AB(u_{1})}Q_{5}(t,A) (t)) ) \biggr\Vert \\& \quad = \frac{u_{1} u_{2}}{AB(u_{1})\Gamma u_{1}} \int _{0}^{t} s^{u_{2}-1}(t-s)^{u_{1}-1} \|Q_{5}\bigl(s,A_{n}(s)\bigr)-Q_{5}(s,A) (s)) \biggl\Vert \,ds \\& \qquad {}+\frac{u_{2}(1-u_{1})t^{u_{2}-1}}{AB(u_{1})} \biggr\Vert Q_{5}\bigl(t,A_{n}(t) \bigr)-Q_{5}(t,A) (t)) \| \\& \quad \leq \biggl(\frac{u_{1} u_{2}}{AB(u_{1})\Gamma u_{1}} \int _{0}^{t} s^{u_{2}-1}(t-s)^{u_{1}-1}+ \frac{u_{2}(1-u_{1})t^{u_{2}-1}}{AB(u_{1})} \biggr)\phi _{5} \Vert A_{n}-A \Vert \\& \quad \leq \biggl( \frac{u_{1} u_{2}\Gamma u_{2}}{AB(u_{1}\Gamma (u_{1}+u_{2}))}+ \frac{u_{2}(1-u_{1})}{AB(u_{1})} \biggr)\phi _{5} \Vert A_{n}-A \Vert \\& \quad \leq \biggl( \frac{u_{1} u_{2}\Gamma u_{2}}{AB(u_{1}\Gamma (u_{1}+u_{2}))}+ \frac{u_{2}(1-u_{1})}{AB(u_{1})} \biggr)^{n} \sigma ^{n} \Vert A_{1}-A \Vert , \end{aligned}$$

where $\sigma < 1$ and as $n \rightarrow \infty $ so $A_{n}\rightarrow A$.

$$\begin{aligned}& \bigl\Vert G_{6}n(t) \bigr\Vert \\& \quad = \bigl\Vert R_{n+1}(t)-R(t) \bigr\Vert \\& \quad = \biggl\Vert \frac{u_{1} u_{2}}{AB(u_{1})\Gamma u_{1}} \int _{0}^{t} s^{u_{2}-1}(t-s)^{u_{1}-1}Q_{6} \bigl(s,R_{n}(s)\bigr)\,ds+ \frac{u_{2}(1-u_{1})t^{u_{2}-1}}{AB(u_{1})}Q_{6} \bigl(t,R_{n}(t)\bigr) \\& \qquad {}- \biggl(\frac{u_{1} u_{2}}{AB(u_{1})\Gamma u_{1}} \int _{0}^{t} s^{u_{2}-1}(t-s)^{u_{1}-1}Q_{6}(s,R) (s)\biggr)\,ds+ \frac{u_{2}(1-u_{1})t^{u_{2}-1}}{AB(u_{1})}Q_{6}(t,R) (t)) ) \biggr\Vert \\& \quad = \frac{u_{1} u_{2}}{AB(u_{1})\Gamma u_{1}} \int _{0}^{t} s^{u_{2}-1}(t-s)^{u_{1}-1} \|Q_{6}\bigl(s,R_{n}(s)\bigr)-Q_{6}(s,R) (s)) \biggl\Vert \,ds \\& \qquad {}+\frac{u_{2}(1-u_{1})t^{u_{2}-1}}{AB(u_{1})} \biggr\Vert Q_{6}\bigl(t,R_{n}(t) \bigr)-Q_{6}(t,R) (t)) \| \\& \quad \leq \biggl(\frac{u_{1} u_{2}}{AB(u_{1})\Gamma u_{1}} \int _{0}^{t} s^{u_{2}-1}(t-s)^{u_{1}-1}+ \frac{u_{2}(1-u_{1})t^{u_{2}-1}}{AB(u_{1})} \biggr)\phi _{6} \Vert R_{n}-R \Vert \\& \quad \leq \biggl( \frac{u_{1} u_{2}\Gamma u_{2}}{AB(u_{1}\Gamma (u_{1}+u_{2}))}+ \frac{u_{2}(1-u_{1})}{AB(u_{1})} \biggr)\phi _{6} \Vert R_{n}-R \Vert \\& \quad \leq \biggl( \frac{u_{1} u_{2}\Gamma u_{2}}{AB(u_{1}\Gamma (u_{1}+u_{2}))}+ \frac{u_{2}(1-u_{1})}{AB(u_{1})} \biggr)^{n} \sigma ^{n} \Vert R_{1}-R \Vert , \end{aligned}$$

where $\sigma < 1$ and as $n \rightarrow \infty $ so $R_{n}\rightarrow R$. Thus we find that $G_{i}n(t)\rightarrow 0$ as $n\rightarrow \infty $ for $i\in N_{1}^{6}$ AND $\sigma < 1$. Hence this completes the proof. □

### Uniqueness of the solution

#### Theorem 2.3

*The fractal fractional model* (1) *has a unique solution if the following inequalities hold true*:

8 $$ \biggl(\frac{u_{1} u_{2}\Gamma u_{2}}{AB(u_{1}\Gamma (u_{1}+u_{2}))}+ \frac{u_{2} (1-u_{1})}{AB(u_{1})} \biggr)\phi _{i}\leq 1,\quad i\in N_{1}^{6}.$$

#### Proof

Let us consider the contradiction that there exists another solution of fractal fractional model (1) such that $S^{*}$, $E^{*}$, $I_{s}^{*}$, $I_{h}^{*}$, $A^{*}$, $R^{*}$ satisfying the given model. We have

$$\begin{aligned}& S^{*}(t)=\frac{u_{1} u_{2}}{AB(u_{1})\Gamma u_{1}} \int _{0}^{t} s^{u_{2}-1}(t-s)^{u_{1}-1}Q_{1} \bigl(s,S^{*}(s)\bigr)\,ds+ \frac{u_{2}(1-u_{1})t^{u_{2}-1}}{AB(u_{1})}Q_{1} \bigl(t,S^{*}(t)\bigr), \\& E^{*}(t)=\frac{u_{1} u_{2}}{AB(u_{1})\Gamma u_{1}} \int _{0}^{t} s^{u_{2}-1}(t-s)^{u_{1}-1}Q_{2} \bigl(s,E^{*}(s)\bigr)\,ds+ \frac{u_{2}(1-u_{1})t^{u_{2}-1}}{AB(u_{1})}Q_{2} \bigl(t,E^{*}(t)\bigr), \\& I_{s}^{*}(t)=\frac{u_{1} u_{2}}{AB(u_{1})\Gamma u_{1}} \int _{0}^{t} s^{u_{2}-1}(t-s)^{u_{1}-1}Q_{3} \bigl(s,I_{s}^{*}(s)\bigr)\,ds+ \frac{u_{2}(1-u_{1})t^{u_{2}-1}}{AB(u_{1})}Q_{3} \bigl(t,I_{s}^{*}(t)\bigr), \\& I_{h}^{*}(t)=\frac{u_{1} u_{2}}{AB(u_{1})\Gamma u_{1}} \int _{0}^{t} s^{u_{2}-1}(t-s)^{u_{1}-1}Q_{4} \bigl(s,I_{h}^{*}(s)\bigr)\,ds+ \frac{u_{2}(1-u_{1})t^{u_{2}-1}}{AB(u_{1})}Q_{4} \bigl(t,I_{h}^{*}(t)\bigr), \\& A^{*}(t)=\frac{u_{1} u_{2}}{AB(u_{1})\Gamma u_{1}} \int _{0}^{t} s^{u_{2}-1}(t-s)^{u_{1}-1}Q_{5} \bigl(s,A^{*}(s)\bigr)\,ds+ \frac{u_{2}(1-u_{1})t^{u_{2}-1}}{AB(u_{1})}Q_{5} \bigl(t,A^{*}(t)\bigr), \\& R^{*}(t)=\frac{u_{1} u_{2}}{AB(u_{1})\Gamma u_{1}} \int _{0}^{t} s^{u_{2}-1}(t-s)^{u_{1}-1}Q_{6} \bigl(s,R^{*}(s)\bigr)\,ds+ \frac{u_{2}(1-u_{1})t^{u_{2}-1}}{AB(u_{1})}Q_{6} \bigl(t,R^{*}(t)\bigr). \end{aligned}$$

Now, taking norm of the difference of $S(t)$, $S^{*}(t)$, we have

$$\begin{aligned}& \bigl\Vert S(t)-S^{*}(t) \bigr\Vert \\& \quad = \biggl\Vert \biggl( \frac{u_{1} u_{2}}{AB(u_{1})\Gamma u_{1}} \int _{0}^{t} s^{u_{2}-1}(t-s)^{u_{1}-1}Q_{1} \bigl(s,S(s)\bigr)\,ds+ \frac{u_{2}(1-u_{1})t^{u_{2}-1}}{AB(u_{1})}Q_{1}\bigl(t,S(t)\bigr) \biggr) \\& \qquad {}- \biggl(\frac{u_{1} u_{2}}{AB(u_{1})\Gamma u_{1}} \int _{0}^{t} s^{u_{2}-1}(t-s)^{u_{1}-1}Q_{1} \bigl(s,S^{*}(s)\bigr)\,ds+ \frac{u_{2}(1-u_{1})t^{u_{2}-1}}{AB(u_{1})}Q_{1} \bigl(t,S^{*}(t)\bigr) \biggr) \biggr\Vert \\& \quad = \frac{u_{1} u_{2}}{AB(u_{1})\Gamma u_{1}} \int _{0}^{t} s^{u_{2}-1}(t-s)^{u_{1}-1} \|Q_{1}(s, S(s)-Q_{1}\bigl(s, S^{*}(s)\bigr) \biggl\Vert \,ds \\& \qquad {}+\frac{u_{2}(1-u_{1})t^{u_{2}-1}}{AB(u_{1})} \biggr\Vert Q_{1}(t, S(t)-Q_{1} \bigl(t, S^{*}(t)\bigr)\|\,ds \\& \quad \leq \biggl(\frac{u_{1} u_{2}}{AB(u_{1})\Gamma u_{1}} \int _{0}^{t} s^{u_{2}-1}(t-s)^{u_{1}-1}+ \frac{u_{2}(1-u_{1})t^{u_{2}-1}}{AB(u_{1})} \biggr)\phi _{1} \bigl\Vert S-S^{*} \bigr\Vert \\& \qquad {}\times \biggl[1- \biggl( \frac{u_{1} u_{2}\Gamma u_{2}}{AB(u_{1}\Gamma (u_{1}+u_{2}))}+ \frac{u_{2}(1-u_{1})}{AB(u_{1})} \biggr)\phi _{1} \biggr] \bigl\Vert S-S^{*} \bigr\Vert \leq 0. \end{aligned}$$

The above inequality is true if $\|S-S^{*}\|=0$, which implies $S=S^{*}$. Similarly, taking norm of the difference of $E(t)$, $E^{*}(t)$, we have

$$\begin{aligned}& \bigl\Vert E(t)-E^{*}(t) \bigr\Vert \\& \quad = \biggl\Vert \biggl( \frac{u_{1} u_{2}}{AB(u_{1})\Gamma u_{1}} \int _{0}^{t} s^{u_{2}-1}(t-s)^{u_{1}-1}Q_{2} \bigl(s,E(s)\bigr)\,ds+ \frac{u_{2}(1-u_{1})t^{u_{2}-1}}{AB(u_{1})}Q_{2}\bigl(t,E(t)\bigr) \biggr) \\& \qquad {}- \biggl(\frac{u_{1} u_{2}}{AB(u_{1})\Gamma u_{1}} \int _{0}^{t} s^{u_{2}-1}(t-s)^{u_{1}-1}Q_{2} \bigl(s,E^{*}(s)\bigr)\,ds+ \frac{u_{2}(1-u_{1})t^{u_{2}-1}}{AB(u_{1})}Q_{2} \bigl(t,E^{*}(t)\bigr) \biggr) \biggr\Vert \\& \quad = \frac{u_{1} u_{2}}{AB(u_{1})\Gamma u_{1}} \int _{0}^{t} s^{u_{2}-1}(t-s)^{u_{1}-1} \|Q_{2}(s, E(s)-Q_{2}\bigl(s, E^{*}(s)\bigr) \biggl\Vert \,ds \\& \qquad {}+\frac{u_{2}(1-u_{1})t^{u_{2}-1}}{AB(u_{1})} \biggr\Vert Q_{2}(t, E(t)-Q_{2} \bigl(t, E^{*}(t)\bigr)\|\,ds \\& \quad \leq \biggl(\frac{u_{1} u_{2}}{AB(u_{1})\Gamma u_{1}} \int _{0}^{t} s^{u_{2}-1}(t-s)^{u_{1}-1}+ \frac{u_{2}(1-u_{1})t^{u_{2}-1}}{AB(u_{1})} \biggr)\phi _{2} \bigl\Vert E-E^{*} \bigr\Vert \\& \qquad {}\times \biggl[1- \biggl( \frac{u_{1} u_{2}\Gamma u_{2}}{AB(u_{1}\Gamma (u_{1}+u_{2}))}+ \frac{u_{2}(1-u_{1})}{AB(u_{1})} \biggr)\phi _{2} \biggr] \bigl\Vert E-E^{*} \bigr\Vert \leq 0. \end{aligned}$$

The above inequality is true if $\|E-E^{*}\|=0$, which implies $E=E^{*}$. Similarly, taking norm of the difference of $I_{s}(t)$, $I_{s}^{*}(t)$, we have

$$\begin{aligned}& \bigl\Vert I_{s}(t)-I_{s}^{*}(t) \bigr\Vert \\& \quad = \biggl\Vert \biggl( \frac{u_{1} u_{2}}{AB(u_{1})\Gamma u_{1}} \int _{0}^{t} s^{u_{2}-1}(t-s)^{u_{1}-1}Q_{3} \bigl(s,I_{s}(s)\bigr)\,ds+ \frac{u_{2}(1-u_{1})t^{u_{2}-1}}{AB(u_{1})}Q_{3} \bigl(t,I_{s}(t)\bigr) \biggr) \\& \qquad {}- \biggl(\frac{u_{1} u_{2}}{AB(u_{1})\Gamma u_{1}} \int _{0}^{t} s^{u_{2}-1}(t-s)^{u_{1}-1}Q_{3} \bigl(s,I_{s}^{*}(s)\bigr)\,ds+ \frac{u_{2}(1-u_{1})t^{u_{2}-1}}{AB(u_{1})}Q_{3} \bigl(t,I_{s}^{*}(t)\bigr) \biggr) \biggr\Vert \\& \quad = \frac{u_{1} u_{2}}{AB(u_{1})\Gamma u_{1}} \int _{0}^{t} s^{u_{2}-1}(t-s)^{u_{1}-1} \|Q_{3}(s, I_{s}(s)-Q_{3}\bigl(s, I_{s}^{*}(s)\bigr) \biggl\Vert \,ds \\& \qquad {}+\frac{u_{2}(1-u_{1})t^{u_{2}-1}}{AB(u_{1})} \biggr\Vert Q_{3}(t, I_{s}(t)-Q_{3} \bigl(t, I_{s}^{*}(t)\bigr)\|\,ds \\& \quad \leq \biggl(\frac{u_{1} u_{2}}{AB(u_{1})\Gamma u_{1}} \int _{0}^{t} s^{u_{2}-1}(t-s)^{u_{1}-1}+ \frac{u_{2}(1-u_{1})t^{u_{2}-1}}{AB(u_{1})} \biggr)\phi _{3} \bigl\Vert I_{s}-I_{s}^{*} \bigr\Vert \\& \qquad {}\times \biggl[1- \biggl( \frac{u_{1} u_{2}\Gamma u_{2}}{AB(u_{1}\Gamma (u_{1}+u_{2}))}+ \frac{u_{2}(1-u_{1})}{AB(u_{1})} \biggr)\phi _{3} \biggr] \bigl\Vert I_{s}-I_{s}^{*} \bigr\Vert \leq 0. \end{aligned}$$

The above inequality is true if $\|I_{s}-I_{s}^{*}\|=0$, which implies $I_{s}=I_{s}^{*}$. Similarly, taking norm of the difference of $I_{h}(t)$, $I_{h}^{*}(t)$, we have

$$\begin{aligned}& \bigl\Vert I_{h}(t)-I_{h}^{*}(t) \bigr\Vert \\& \quad = \biggl\Vert \biggl( \frac{u_{1} u_{2}}{AB(u_{1})\Gamma u_{1}} \int _{0}^{t} s^{u_{2}-1}(t-s)^{u_{1}-1}Q_{4} \bigl(s,I_{h}(s)\bigr)\,ds+ \frac{u_{2}(1-u_{1})t^{u_{2}-1}}{AB(u_{1})}Q_{4} \bigl(t,I_{h}(t)\bigr) \biggr) \\& \qquad {}- \biggl(\frac{u_{1} u_{2}}{AB(u_{1})\Gamma u_{1}} \int _{0}^{t} s^{u_{2}-1}(t-s)^{u_{1}-1}Q_{4} \bigl(s,I_{h}^{*}(s)\bigr)\,ds+ \frac{u_{2}(1-u_{1})t^{u_{2}-1}}{AB(u_{1})}Q_{4} \bigl(t,I_{h}^{*}(t)\bigr) \biggr) \biggr\Vert \\& \quad = \frac{u_{1} u_{2}}{AB(u_{1})\Gamma u_{1}} \int _{0}^{t} s^{u_{2}-1}(t-s)^{u_{1}-1} \|Q_{4}(s, I_{h}(s)-Q_{4}\bigl(s, I_{h}^{*}(s)\bigr) \biggl\Vert \,ds \\& \qquad {}+\frac{u_{2}(1-u_{1})t^{u_{2}-1}}{AB(u_{1})} \biggr\Vert Q_{4}(t, I_{h}(t)-Q_{4} \bigl(t, I_{h}^{*}(t)\bigr)\|\,ds \\& \quad \leq \biggl(\frac{u_{1} u_{2}}{AB(u_{1})\Gamma u_{1}} \int _{0}^{t} s^{u_{2}-1}(t-s)^{u_{1}-1}+ \frac{u_{2}(1-u_{1})t^{u_{2}-1}}{AB(u_{1})} \biggr)\phi _{4} \bigl\Vert I_{h}-I_{h}^{*} \bigr\Vert \\& \qquad {}\times \biggl[1- \biggl( \frac{u_{1} u_{2}\Gamma u_{2}}{AB(u_{1}\Gamma (u_{1}+u_{2}))}+ \frac{u_{2}(1-u_{1})}{AB(u_{1})} \biggr)\phi _{4} \biggr] \bigl\Vert I_{h}-I_{h}^{*} \bigr\Vert \leq 0. \end{aligned}$$

The above inequality is true if $\|I_{h}-I_{h}^{*}\|=0$, which implies $I_{h}=I_{h}^{*}$. Similarly, taking norm of the difference of $A(t)$, $A^{*}(t)$, we have

$$\begin{aligned}& \bigl\Vert A(t)-A^{*}(t) \bigr\Vert \\& \quad = \biggl\Vert \biggl( \frac{u_{1} u_{2}}{AB(u_{1})\Gamma u_{1}} \int _{0}^{t} s^{u_{2}-1}(t-s)^{u_{1}-1}Q_{5} \bigl(s,A(s)\bigr)\,ds+ \frac{u_{2}(1-u_{1})t^{u_{2}-1}}{AB(u_{1})}Q_{5}\bigl(t,A(t)\bigr) \biggr) \\& \qquad {}- \biggl(\frac{u_{1} u_{2}}{AB(u_{1})\Gamma u_{1}} \int _{0}^{t} s^{u_{2}-1}(t-s)^{u_{1}-1}Q_{5} \bigl(s,A^{*}(s)\bigr)\,ds \\& \qquad {}+ \frac{u_{2}(1-u_{1})t^{u_{2}-1}}{AB(u_{1})}Q_{5} \bigl(t,A^{*}(t)\bigr) \biggr) \biggr\Vert \\& \quad = \frac{u_{1} u_{2}}{AB(u_{1})\Gamma u_{1}} \int _{0}^{t} s^{u_{2}-1}(t-s)^{u_{1}-1} \|Q_{5}(s, A(s)-Q_{5}\bigl(s, A^{*}(s)\bigr) \biggl\Vert \,ds \\& \qquad {}+\frac{u_{2}(1-u_{1})t^{u_{2}-1}}{AB(u_{1})} \biggr\Vert Q_{5}(t, A(t)-Q_{5} \bigl(t, A^{*}(t)\bigr)\|\,ds \\& \quad \leq \biggl(\frac{u_{1} u_{2}}{AB(u_{1})\Gamma u_{1}} \int _{0}^{t} s^{u_{2}-1}(t-s)^{u_{1}-1}+ \frac{u_{2}(1-u_{1})t^{u_{2}-1}}{AB(u_{1})} \biggr)\phi _{5} \bigl\Vert A-A^{*} \bigr\Vert \\& \qquad {}\times \biggl[1- \biggl( \frac{u_{1} u_{2}\Gamma u_{2}}{AB(u_{1}\Gamma (u_{1}+u_{2}))}+ \frac{u_{2}(1-u_{1})}{AB(u_{1})} \biggr)\phi _{5} \biggr] \bigl\Vert A-A^{*} \bigr\Vert \leq 0. \end{aligned}$$

The above inequality is true if $\|A-A^{*}\|=0$, which implies $A=A^{*}$. Similarly, taking norm of the difference of $R(t)$, $R^{*}(t)$, we have

$$\begin{aligned}& \bigl\Vert R(t)-R^{*}(t) \bigr\Vert \\& \quad = \biggl\Vert \biggl( \frac{u_{1} u_{2}}{AB(u_{1})\Gamma u_{1}} \int _{0}^{t} s^{u_{2}-1}(t-s)^{u_{1}-1}Q_{6} \bigl(s,R(s)\bigr)\,ds+ \frac{u_{2}(1-u_{1})t^{u_{2}-1}}{AB(u_{1})}Q_{6}\bigl(t,R(t)\bigr) \biggr) \\& \qquad {}- \biggl(\frac{u_{1} u_{2}}{AB(u_{1})\Gamma u_{1}} \int _{0}^{t} s^{u_{2}-1}(t-s)^{u_{1}-1}Q_{6} \bigl(s,R^{*}(s)\bigr)\,ds+ \frac{u_{2}(1-u_{1})t^{u_{2}-1}}{AB(u_{1})}Q_{6} \bigl(t,R^{*}(t)\bigr) \biggr) \biggr\Vert \\& \quad = \frac{u_{1} u_{2}}{AB(u_{1})\Gamma u_{1}} \int _{0}^{t} s^{u_{2}-1}(t-s)^{u_{1}-1} \|Q_{6}(s, R(s)-Q_{2}\bigl(s, R^{*}(s)\bigr) \biggl\Vert \,ds \\& \qquad {}+\frac{u_{2}(1-u_{1})t^{u_{2}-1}}{AB(u_{1})} \biggr\Vert Q_{6}(t, R(t)-Q_{6} \bigl(t, R^{*}(t)\bigr)\|\,ds \\& \quad \leq \biggl(\frac{u_{1} u_{2}}{AB(u_{1})\Gamma u_{1}} \int _{0}^{t} s^{u_{2}-1}(t-s)^{u_{1}-1}+ \frac{u_{2}(1-u_{1})t^{u_{2}-1}}{AB(u_{1})} \biggr)\phi _{6} \bigl\Vert R-R^{*} \bigr\Vert \\& \qquad {}\times \biggl[1- \biggl( \frac{u_{1} u_{2}\Gamma u_{2}}{AB(u_{1}\Gamma (u_{1}+u_{2}))}+ \frac{u_{2}(1-u_{1})}{AB(u_{1})} \biggr)\phi _{6} \biggr] \bigl\Vert R-R^{*} \bigr\Vert \leq 0. \end{aligned}$$

The above inequality is true if $\|R-R^{*}\|=0$, which implies $R(t)$, $R^{*}(t)$. Hence we see that $S=S^{*}$, $E=E^{*}$, $I_{s}=I_{s}^{*}$, $rI_{h}=I_{h}^{*}$, $A=A^{*}$, $R=R^{*}$, so our supposition is wrong and the theorem has a unique solution. □

**Hyers–Ulam stability**

#### Definition 2.4

The fractal fractional integrals (6) are said to be Hyers–Ulam stable if there exist constants $\alpha _{i} > 0$, $i \in N_{1}^{6}$ satisfying, for every $\beta _{i} > 0$, $i \in N_{1}^{6}$, the following:

$$\begin{aligned}& \biggl\vert S(t)-\frac{u_{1} u_{2}}{AB(u_{1})\Gamma u_{1}} \int _{0}^{t} s^{u_{2}-1}(t-s)^{u_{1}-1}Q_{1} \bigl(s,S(s)\bigr)\,ds+ \frac{u_{2}(1-u_{1})t^{u_{2}-1}}{AB(u_{1})}Q_{1}\bigl(t,S(t)\bigr) \biggr\vert \leq \beta _{1}, \\& \biggl\vert E(t)-\frac{u_{1} u_{2}}{AB(u_{1})\Gamma u_{1}} \int _{0}^{t} s^{u_{2}-1}(t-s)^{u_{1}-1}Q_{2} \bigl(s,E(s)\bigr)\,ds+ \frac{u_{2}(1-u_{1})t^{u_{2}-1}}{AB(u_{1})}Q_{2}\bigl(t,E(t)\bigr) \biggr\vert \leq \beta _{2}, \\& \biggl\vert I_{s}(t)-\frac{u_{1} u_{2}}{AB(u_{1})\Gamma u_{1}} \int _{0}^{t} s^{u_{2}-1}(t-s)^{u_{1}-1}Q_{3} \bigl(s,I_{s}(s)\bigr)\,ds+ \frac{u_{2}(1-u_{1})t^{u_{2}-1}}{AB(u_{1})}Q_{3} \bigl(t,I_{s}(t)\bigr) \biggr\vert \\& \quad \leq \beta _{3}, \\& \biggl\vert I_{h}(t)-\frac{u_{1} u_{2}}{AB(u_{1})\Gamma u_{1}} \int _{0}^{t} s^{u_{2}-1}(t-s)^{u_{1}-1}Q_{4} \bigl(s,I_{h}(s)\bigr)\,ds+ \frac{u_{2}(1-u_{1})t^{u_{2}-1}}{AB(u_{1})}Q_{4} \bigl(t,I_{h}(t)\bigr) \biggr\vert \\& \quad \leq \beta _{4}, \\& \biggl\vert A(t)-\frac{u_{1} u_{2}}{AB(u_{1})\Gamma u_{1}} \int _{0}^{t} s^{u_{2}-1}(t-s)^{u_{1}-1}Q_{5} \bigl(s,A(s)\bigr)\,ds+ \frac{u_{2}(1-u_{1})t^{u_{2}-1}}{AB(u_{1})}Q_{5}\bigl(t,A(t)\bigr) \biggr\vert \\& \quad \leq \beta _{5}, \\& \biggl\vert R(t)-\frac{u_{1} u_{2}}{AB(u_{1})\Gamma u_{1}} \int _{0}^{t} s^{u_{2}-1}(t-s)^{u_{1}-1}Q_{6} \bigl(s,R(s)\bigr)\,ds+ \frac{u_{2}(1-u_{1})t^{u_{2}-1}}{AB(u_{1})}Q_{6}\bigl(t,R(t)\bigr) \biggr\vert \leq \beta _{6}. \end{aligned}$$

There exists an approximate solution of model (1) $S^{*}(t)$, $E^{*}(t)$, $I_{s}^{*}(t)$, $I_{h}^{*}(t)$, $A^{*}(t)$, $R^{*}(t)$ that satisfies the given model, such that

$$\begin{aligned}& \bigl\vert S(t)-S^{*}(t) \bigr\vert \\& \quad = \biggl\vert \biggl( \frac{u_{1} u_{2}}{AB(u_{1})\Gamma u_{1}} \int _{0}^{t} s^{u_{2}-1}(t-s)^{u_{1}-1}Q_{1} \bigl(s,S(s)\bigr)\,ds+ \frac{u_{2}(1-u_{1})t^{u_{2}-1}}{AB(u_{1})}Q_{1}\bigl(t,S(t)\bigr) \biggr) \\& \qquad {}- \biggl(\frac{u_{1} u_{2}}{AB(u_{1})\Gamma u_{1}} \int _{0}^{t} s^{u_{2}-1}(t-s)^{u_{1}-1}Q_{1} \bigl(s,S^{*}(s)\bigr)\,ds+ \frac{u_{2}(1-u_{1})t^{u_{2}-1}}{AB(u_{1})}Q_{1} \bigl(t,S^{*}(t)\bigr) \biggr) \biggr\vert \\& \quad = \frac{u_{1} u_{2}}{AB(u_{1})\Gamma u_{1}} \int _{0}^{t} s^{u_{2}-1}(t-s)^{u_{1}-1} \bigl\vert Q_{1}(s, S(s)-Q_{1}\bigl(s, S^{*}(s) \bigr) \bigr\vert \,ds \\& \qquad {}+\frac{u_{2}(1-u_{1})t^{u_{2}-1}}{AB(u_{1})} \bigl\vert Q_{1}(t, S(t)-Q_{1} \bigl(t, S^{*}(t)\bigr) \bigr\vert \,ds \\& \quad \leq \biggl(\frac{u_{1} u_{2}}{AB(u_{1})\Gamma u_{1}} \int _{0}^{t} s^{u_{2}-1}(t-s)^{u_{1}-1}+ \frac{u_{2}(1-u_{1})t^{u_{2}-1}}{AB(u_{1})} \biggr)\phi _{1} \bigl\Vert S-S^{*} \bigr\Vert \\& \quad \leq \biggl( \frac{u_{1} u_{2}\Gamma u_{2}}{AB(u_{1}\Gamma (u_{1}+u_{2}))}+ \frac{u_{2}(1-u_{1})}{AB(u_{1})} \biggr)\phi _{1} \bigl\Vert S-S^{*} \bigr\Vert . \end{aligned}$$

Let $\zeta _{1}= ( \frac{u_{1} u_{2}\Gamma u_{2}}{AB(u_{1}\Gamma (u_{1}+u_{2}))}+ \frac{u_{2}(1-u_{1})}{AB(u_{1})} )\|S-S^{*}\|$, $\eta _{1}=\phi _{1}$,so the above inequality becomes $|S-S^{*}|\leq \zeta _{1} \eta _{1}$.

$$\begin{aligned}& \bigl\vert E(t)-E^{*}(t) \bigr\vert \\& \quad = \biggl\vert \biggl( \frac{u_{1} u_{2}}{AB(u_{1})\Gamma u_{1}} \int _{0}^{t} s^{u_{2}-1}(t-s)^{u_{1}-1}Q_{2} \bigl(s,E(s)\bigr)\,ds+ \frac{u_{2}(1-u_{1})t^{u_{2}-1}}{AB(u_{1})}Q_{2}\bigl(t,E(t)\bigr) \biggr) \\& \qquad {}- \biggl(\frac{u_{1} u_{2}}{AB(u_{1})\Gamma u_{1}} \int _{0}^{t} s^{u_{2}-1}(t-s)^{u_{1}-1}Q_{2} \bigl(s,E^{*}(s)\bigr)\,ds+ \frac{u_{2}(1-u_{1})t^{u_{2}-1}}{AB(u_{1})}Q_{2} \bigl(t,E^{*}(t)\bigr) \biggr) \biggr\vert \\& \quad = \frac{u_{1} u_{2}}{AB(u_{1})\Gamma u_{1}} \int _{0}^{t} s^{u_{2}-1}(t-s)^{u_{1}-1} \bigl\vert Q_{2}(s, E(s)-Q_{2}\bigl(s, E^{*}(s) \bigr) \bigr\vert \,ds \\& \qquad {}+\frac{u_{2}(1-u_{1})t^{u_{2}-1}}{AB(u_{1})} \bigl\vert Q_{2}(t, E(t)-Q_{2} \bigl(t, E^{*}(t)\bigr) \bigr\vert \,ds \\& \quad \leq \biggl(\frac{u_{1} u_{2}}{AB(u_{1})\Gamma u_{1}} \int _{0}^{t} s^{u_{2}-1}(t-s)^{u_{1}-1}+ \frac{u_{2}(1-u_{1})t^{u_{2}-1}}{AB(u_{1})} \biggr)\phi _{2} \bigl\Vert E-E^{*} \bigr\Vert \\& \quad \leq \biggl( \frac{u_{1} u_{2}\Gamma u_{2}}{AB(u_{1}\Gamma (u_{1}+u_{2}))}+ \frac{u_{2}(1-u_{1})}{AB(u_{1})} \biggr)\phi _{2} \bigl\Vert E-E^{*} \bigr\Vert . \end{aligned}$$

Let $\zeta _{2}= ( \frac{u_{1} u_{2}\Gamma u_{2}}{AB(u_{1}\Gamma (u_{1}+u_{2}))}+ \frac{u_{2}(1-u_{1})}{AB(u_{1})} )\|E-E^{*}\|$, $\eta _{2}=\phi _{2}$,so the above inequality becomes $|E-E^{*}|\leq \zeta _{2} \eta _{2}$.

$$\begin{aligned}& \bigl\vert I_{s}(t)-I_{s}^{*}(t) \bigr\vert \\& \quad = \biggl\vert \biggl( \frac{u_{1} u_{2}}{AB(u_{1})\Gamma u_{1}} \int _{0}^{t} s^{u_{2}-1}(t-s)^{u_{1}-1}Q_{3} \bigl(s,I_{s}(s)\bigr)\,ds+ \frac{u_{2}(1-u_{1})t^{u_{2}-1}}{AB(u_{1})}Q_{3} \bigl(t,I_{s}(t)\bigr) \biggr) \\& \qquad {}- \biggl(\frac{u_{1} u_{2}}{AB(u_{1})\Gamma u_{1}} \int _{0}^{t} s^{u_{2}-1}(t-s)^{u_{1}-1}Q_{3} \bigl(s,I_{s}^{*}(s)\bigr)\,ds+ \frac{u_{2}(1-u_{1})t^{u_{2}-1}}{AB(u_{1})}Q_{3} \bigl(t,I_{s}^{*}(t)\bigr) \biggr) \biggr\vert \\& \quad = \frac{u_{1} u_{2}}{AB(u_{1})\Gamma u_{1}} \int _{0}^{t} s^{u_{2}-1}(t-s)^{u_{1}-1} \bigl\vert Q_{3}(s, I_{s}(s)-Q_{3}\bigl(s, I_{s}^{*}(s)\bigr) \bigr\vert \,ds \\& \qquad {}+\frac{u_{2}(1-u_{1})t^{u_{2}-1}}{AB(u_{1})} \bigl\vert Q_{3}(t, I_{s}(t)-Q_{3} \bigl(t, I_{s}^{*}(t)\bigr) \bigr\vert \,ds \\& \quad \leq \biggl( \frac{u_{1} u_{2}\Gamma u_{2}}{AB(u_{1}\Gamma (u_{1}+u_{2}))}+ \frac{u_{2}(1-u_{1})}{AB(u_{1})} \biggr)\phi _{3} \bigl\Vert I_{s}-I_{s}^{*} \bigr\Vert . \end{aligned}$$

Let $\zeta _{3}= ( \frac{u_{1} u_{2}\Gamma u_{2}}{AB(u_{1}\Gamma (u_{1}+u_{2}))}+ \frac{u_{2}(1-u_{1})}{AB(u_{1})} )\|I_{s}-I_{s}^{*}\|$, $\eta _{3}=\phi _{3}$, so the above inequality becomes $|I_{s}-I_{s}^{*}|\leq \zeta _{3} \eta _{3}$.

$$\begin{aligned}& \bigl\vert I_{h}(t)-I_{h}^{*}(t) \bigr\vert \\& \quad = \biggl\vert \biggl( \frac{u_{1} u_{2}}{AB(u_{1})\Gamma u_{1}} \int _{0}^{t} s^{u_{2}-1}(t-s)^{u_{1}-1}Q_{4} \bigl(s,I_{h}(s)\bigr)\,ds+ \frac{u_{2}(1-u_{1})t^{u_{2}-1}}{AB(u_{1})}Q_{4} \bigl(t,I_{h}(t)\bigr) \biggr) \\& \qquad {}- \biggl(\frac{u_{1} u_{2}}{AB(u_{1})\Gamma u_{1}} \int _{0}^{t} s^{u_{2}-1}(t-s)^{u_{1}-1}Q_{4} \bigl(s,I_{h}^{*}(s)\bigr)\,ds+ \frac{u_{2}(1-u_{1})t^{u_{2}-1}}{AB(u_{1})}Q_{4} \bigl(t,I_{h}^{*}(t)\bigr) \biggr) \biggr\vert \\& \quad = \frac{u_{1} u_{2}}{AB(u_{1})\Gamma u_{1}} \int _{0}^{t} s^{u_{2}-1}(t-s)^{u_{1}-1} \bigl\vert Q_{4}(s, I_{h}(s)-Q_{4}\bigl(s, I_{h}^{*}(s)\bigr) \bigr\vert \,ds \\& \qquad {}+\frac{u_{2}(1-u_{1})t^{u_{2}-1}}{AB(u_{1})} \bigl\vert Q_{4}(t, I_{h}(t)-Q_{4} \bigl(t, I_{h}^{*}(t)\bigr) \bigr\vert \,ds \\& \quad \leq \biggl(\frac{u_{1} u_{2}}{AB(u_{1})\Gamma u_{1}} \int _{0}^{t} s^{u_{2}-1}(t-s)^{u_{1}-1}+ \frac{u_{2}(1-u_{1})t^{u_{2}-1}}{AB(u_{1})} \biggr)\phi _{4} \bigl\Vert I_{h}-I_{h}^{*} \bigr\Vert \\& \quad \leq \biggl( \frac{u_{1} u_{2}\Gamma u_{2}}{AB(u_{1}\Gamma (u_{1}+u_{2}))}+ \frac{u_{2}(1-u_{1})}{AB(u_{1})} \biggr)\phi _{4} \bigl\Vert I_{h}-I_{h}^{*} \bigr\Vert . \end{aligned}$$

Let $\zeta _{4}= ( \frac{u_{1} u_{2}\Gamma u_{2}}{AB(u_{1}\Gamma (u_{1}+u_{2}))}+ \frac{u_{2}(1-u_{1})}{AB(u_{1})} )\|I_{h}-I_{h}^{*}\|$, $\eta _{4}=\phi _{4}$, so the above inequality becomes $|I_{h}-I_{h}^{*}|\leq \zeta _{4} \eta _{4}$.

$$\begin{aligned}& \bigl\vert A(t)-A^{*}(t) \bigr\vert \\& \quad = \biggl\vert \biggl( \frac{u_{1} u_{2}}{AB(u_{1})\Gamma u_{1}} \int _{0}^{t} s^{u_{2}-1}(t-s)^{u_{1}-1}Q_{5} \bigl(s,A(s)\bigr)\,ds+ \frac{u_{2}(1-u_{1})t^{u_{2}-1}}{AB(u_{1})}Q_{5}\bigl(t,A(t)\bigr) \biggr) \\& \qquad {}- \biggl(\frac{u_{1} u_{2}}{AB(u_{1})\Gamma u_{1}} \int _{0}^{t} s^{u_{2}-1}(t-s)^{u_{1}-1}Q_{5} \bigl(s,A^{*}(s)\bigr)\,ds+ \frac{u_{2}(1-u_{1})t^{u_{2}-1}}{AB(u_{1})}Q_{5} \bigl(t,A^{*}(t)\bigr) \biggr) \biggr\vert \\& \quad = \frac{u_{1} u_{2}}{AB(u_{1})\Gamma u_{1}} \int _{0}^{t} s^{u_{2}-1}(t-s)^{u_{1}-1} \bigl\vert Q_{5}(s, A(s)-Q_{5}\bigl(s, A^{*}(s) \bigr) \bigr\vert \,ds \\& \qquad {}+\frac{u_{2}(1-u_{1})t^{u_{2}-1}}{AB(u_{1})} \bigl\vert Q_{5}(t, A(t)-Q_{5} \bigl(t, A^{*}(t)\bigr) \bigr\vert \,ds \\& \quad \leq \biggl( \frac{u_{1} u_{2}\Gamma u_{2}}{AB(u_{1}\Gamma (u_{1}+u_{2}))}+ \frac{u_{2}(1-u_{1})}{AB(u_{1})} \biggr)\phi _{5} \bigl\Vert A-A^{*} \bigr\Vert . \end{aligned}$$

Let $\zeta _{5}= ( \frac{u_{1} u_{2}\Gamma u_{2}}{AB(u_{1}\Gamma (u_{1}+u_{2}))}+ \frac{u_{2}(1-u_{1})}{AB(u_{1})} )\|A-A^{*}\|$, $\eta _{5}=\phi _{5}$, so the above inequality becomes $|A-A^{*}|\leq \zeta _{5} \eta _{5}$.

$$\begin{aligned}& \bigl\vert R(t)-R^{*}(t) \bigr\vert \\& \quad = \biggl\vert \biggl( \frac{u_{1} u_{2}}{AB(u_{1})\Gamma u_{1}} \int _{0}^{t} s^{u_{2}-1}(t-s)^{u_{1}-1}Q_{6} \bigl(s,R(s)\bigr)\,ds+ \frac{u_{2}(1-u_{1})t^{u_{2}-1}}{AB(u_{1})}Q_{6}\bigl(t,R(t)\bigr) \biggr) \\& \qquad {}- \biggl(\frac{u_{1} u_{2}}{AB(u_{1})\Gamma u_{1}} \int _{0}^{t} s^{u_{2}-1}(t-s)^{u_{1}-1}Q_{6} \bigl(s,R^{*}(s)\bigr)\,ds+ \frac{u_{2}(1-u_{1})t^{u_{2}-1}}{AB(u_{1})}Q_{6} \bigl(t,R^{*}(t)\bigr) \biggr) \biggr\vert \\& \quad = \frac{u_{1} u_{2}}{AB(u_{1})\Gamma u_{1}} \int _{0}^{t} s^{u_{2}-1}(t-s)^{u_{1}-1}|Q_{6}(s, R(s)-Q_{6}\bigl(s, R^{*}(s)\bigr) \biggl\vert \,ds \\& \qquad {}+\frac{u_{2}(1-u_{1})t^{u_{2}-1}}{AB(u_{1})} \biggr\vert Q_{6}(t, R(t)-Q_{6} \bigl(t, R^{*}(t)\bigr)|\,ds \\& \quad \leq \biggl( \frac{u_{1} u_{2}\Gamma u_{2}}{AB(u_{1}\Gamma (u_{1}+u_{2}))}+ \frac{u_{2}(1-u_{1})}{AB(u_{1})} \biggr)\phi _{6} \bigl\Vert R-R^{*} \bigr\Vert . \end{aligned}$$

Let $\zeta _{6}= ( \frac{u_{1} u_{2}\Gamma u_{2}}{AB(u_{1}\Gamma (u_{1}+u_{2}))}+ \frac{u_{2}(1-u_{1})}{AB(u_{1})} )\|R-R^{*}\|$, $\eta _{6}=\phi _{6}$, so the above inequality becomes $|R-R^{*}|\leq \zeta _{6} \eta _{6}$.

#### Theorem 2.5

*With assumption* (G*), *the fractal fractional model* (1) *is Hyers–Ulam stable*.

#### Proof

We know that the fractal fractional model (1) has a unique solution. Let there exist an approximate solution of model (1) $S^{*}(t)$, $E^{*}(t)$, $I_{s}^{*}(t)$, $I_{h}^{*}(t)$, $A^{*}(t)$, $R^{*}(t)$ that satisfies the given model, such that

$$\begin{aligned}& \bigl\vert S(t)-S^{*}(t) \bigr\vert \\& \quad = \biggl\vert \biggl( \frac{u_{1} u_{2}}{AB(u_{1})\Gamma u_{1}} \int _{0}^{t} s^{u_{2}-1}(t-s)^{u_{1}-1}Q_{1} \bigl(s,S(s)\bigr)\,ds+ \frac{u_{2}(1-u_{1})t^{u_{2}-1}}{AB(u_{1})}Q_{1}\bigl(t,S(t)\bigr) \biggr) \\& \qquad {}- \biggl(\frac{u_{1} u_{2}}{AB(u_{1})\Gamma u_{1}} \int _{0}^{t} s^{u_{2}-1}(t-s)^{u_{1}-1}Q_{1} \bigl(s,S^{*}(s)\bigr)\,ds+ \frac{u_{2}(1-u_{1})t^{u_{2}-1}}{AB(u_{1})}Q_{1} \bigl(t,S^{*}(t)\bigr) \biggr) \biggr\vert \\& \quad = \frac{u_{1} u_{2}}{AB(u_{1})\Gamma u_{1}} \int _{0}^{t} s^{u_{2}-1}(t-s)^{u_{1}-1} \bigl\vert Q_{1}(s, S(s)-Q_{1}\bigl(s, S^{*}(s) \bigr) \bigr\vert \,ds \\& \qquad {}+\frac{u_{2}(1-u_{1})t^{u_{2}-1}}{AB(u_{1})} \bigl\vert Q_{1}(t, S(t)-Q_{1} \bigl(t, S^{*}(t)\bigr) \bigr\vert \,ds \\& \quad \leq \biggl(\frac{u_{1} u_{2}}{AB(u_{1})\Gamma u_{1}} \int _{0}^{t} s^{u_{2}-1}(t-s)^{u_{1}-1}+ \frac{u_{2}(1-u_{1})t^{u_{2}-1}}{AB(u_{1})} \biggr)\phi _{1} \bigl\Vert S-S^{*} \bigr\Vert \\& \quad \leq \biggl( \frac{u_{1} u_{2}\Gamma u_{2}}{AB(u_{1}\Gamma (u_{1}+u_{2}))}+ \frac{u_{2}(1-u_{1})}{AB(u_{1})} \biggr)\phi _{1} \bigl\Vert S-S^{*} \bigr\Vert . \end{aligned}$$

Let $\alpha _{1}= ( \frac{u_{1} u_{2}\Gamma u_{2}}{AB(u_{1}\Gamma (u_{1}+u_{2}))}+ \frac{u_{2}(1-u_{1})}{AB(u_{1})} )\|S-S^{*}\|$, $\Delta _{1}=\phi _{1}$, so the above inequality becomes $|S-S^{*}|\leq \alpha _{1} \Delta _{1}$.

$$\begin{aligned}& \bigl\vert E(t)-E^{*}(t) \bigr\vert \\& \quad = \biggl\vert \biggl( \frac{u_{1} u_{2}}{AB(u_{1})\Gamma u_{1}} \int _{0}^{t} s^{u_{2}-1}(t-s)^{u_{1}-1}Q_{2} \bigl(s,E(s)\bigr)\,ds+ \frac{u_{2}(1-u_{1})t^{u_{2}-1}}{AB(u_{1})}Q_{2}\bigl(t,E(t)\bigr) \biggr) \\& \qquad {}- \biggl(\frac{u_{1} u_{2}}{AB(u_{1})\Gamma u_{1}} \int _{0}^{t} s^{u_{2}-1}(t-s)^{u_{1}-1}Q_{2} \bigl(s,E^{*}(s)\bigr)\,ds \\& \qquad {}+ \frac{u_{2}(1-u_{1})t^{u_{2}-1}}{AB(u_{1})}Q_{2} \bigl(t,E^{*}(t)\bigr) \biggr) \biggr\vert \\& \quad = \frac{u_{1} u_{2}}{AB(u_{1})\Gamma u_{1}} \int _{0}^{t} s^{u_{2}-1}(t-s)^{u_{1}-1} \bigl\vert Q_{2}(s, E(s)-Q_{2}\bigl(s, E^{*}(s) \bigr) \bigr\vert \,ds \\& \qquad {}+\frac{u_{2}(1-u_{1})t^{u_{2}-1}}{AB(u_{1})} \bigl\vert Q_{2}(t, E(t)-Q_{2} \bigl(t, E^{*}(t)\bigr) \bigr\vert \,ds \\& \quad \leq \biggl(\frac{u_{1} u_{2}}{AB(u_{1})\Gamma u_{1}} \int _{0}^{t} s^{u_{2}-1}(t-s)^{u_{1}-1}+ \frac{u_{2}(1-u_{1})t^{u_{2}-1}}{AB(u_{1})} \biggr)\phi _{2} \bigl\Vert E-E^{*} \bigr\Vert \\& \quad \leq \biggl( \frac{u_{1} u_{2}\Gamma u_{2}}{AB(u_{1}\Gamma (u_{1}+u_{2}))}+ \frac{u_{2}(1-u_{1})}{AB(u_{1})} \biggr)\phi _{2} \bigl\Vert E-E^{*} \bigr\Vert . \end{aligned}$$

Let $\alpha _{2}= ( \frac{u_{1} u_{2}\Gamma u_{2}}{AB(u_{1}\Gamma (u_{1}+u_{2}))}+ \frac{u_{2}(1-u_{1})}{AB(u_{1})} )\|E-E^{*}\|$, $\Delta _{2}=\phi _{2}$, so the above inequality becomes $|E-E^{*}|\leq \alpha _{2} \Delta _{2}$.

$$\begin{aligned}& \bigl\vert I_{s}(t)-I_{s}^{*}(t) \bigr\vert \\& \quad = \biggl\vert \biggl( \frac{u_{1} u_{2}}{AB(u_{1})\Gamma u_{1}} \int _{0}^{t} s^{u_{2}-1}(t-s)^{u_{1}-1}Q_{3} \bigl(s,I_{s}(s)\bigr)\,ds+ \frac{u_{2}(1-u_{1})t^{u_{2}-1}}{AB(u_{1})}Q_{3} \bigl(t,I_{s}(t)\bigr) \biggr) \\& \qquad {}- \biggl(\frac{u_{1} u_{2}}{AB(u_{1})\Gamma u_{1}} \int _{0}^{t} s^{u_{2}-1}(t-s)^{u_{1}-1}Q_{3} \bigl(s,I_{s}^{*}(s)\bigr)\,ds \\& \qquad {}+ \frac{u_{2}(1-u_{1})t^{u_{2}-1}}{AB(u_{1})}Q_{3} \bigl(t,I_{s}^{*}(t)\bigr) \biggr) \biggr\vert \\& \quad = \frac{u_{1} u_{2}}{AB(u_{1})\Gamma u_{1}} \int _{0}^{t} s^{u_{2}-1}(t-s)^{u_{1}-1} \bigl\vert Q_{3}(s, I_{s}(s)-Q_{3}\bigl(s, I_{s}^{*}(s)\bigr) \bigr\vert \,ds \\& \qquad {}+\frac{u_{2}(1-u_{1})t^{u_{2}-1}}{AB(u_{1})} \bigl\vert Q_{3}(t, I_{s}(t)-Q_{3} \bigl(t, I_{s}^{*}(t)\bigr) \bigr\vert \,ds \\& \quad \leq \biggl(\frac{u_{1} u_{2}}{AB(u_{1})\Gamma u_{1}} \int _{0}^{t} s^{u_{2}-1}(t-s)^{u_{1}-1}+ \frac{u_{2}(1-u_{1})t^{u_{2}-1}}{AB(u_{1})} \biggr)\phi _{3} \bigl\Vert I_{s}-I_{s}^{*} \bigr\Vert \\& \quad \leq \biggl( \frac{u_{1} u_{2}\Gamma u_{2}}{AB(u_{1}\Gamma (u_{1}+u_{2}))}+ \frac{u_{2}(1-u_{1})}{AB(u_{1})} \biggr)\phi _{3} \bigl\Vert I_{s}-I_{s}^{*} \bigr\Vert . \end{aligned}$$

Let $\alpha _{3}= ( \frac{u_{1} u_{2}\Gamma u_{2}}{AB(u_{1}\Gamma (u_{1}+u_{2}))}+ \frac{u_{2}(1-u_{1})}{AB(u_{1})} )\|I_{s}-I_{s}^{*}\|$, $\Delta _{3}=\phi _{3}$, so the above inequality becomes $|I_{s}-I_{s}^{*}|\leq \alpha _{3} \Delta _{3}$.

$$\begin{aligned}& \bigl\vert I_{h}(t)-I_{h}^{*}(t) \bigr\vert \\& \quad = \biggl\vert \biggl( \frac{u_{1} u_{2}}{AB(u_{1})\Gamma u_{1}} \int _{0}^{t} s^{u_{2}-1}(t-s)^{u_{1}-1}Q_{4} \bigl(s,I_{h}(s)\bigr)\,ds+ \frac{u_{2}(1-u_{1})t^{u_{2}-1}}{AB(u_{1})}Q_{4} \bigl(t,I_{h}(t)\bigr) \biggr) \\& \qquad {}- \biggl(\frac{u_{1} u_{2}}{AB(u_{1})\Gamma u_{1}} \int _{0}^{t} s^{u_{2}-1}(t-s)^{u_{1}-1}Q_{4} \bigl(s,I_{h}^{*}(s)\bigr)\,ds \\& \qquad {}+ \frac{u_{2}(1-u_{1})t^{u_{2}-1}}{AB(u_{1})}Q_{4} \bigl(t,I_{h}^{*}(t)\bigr) \biggr) \biggr\vert \\& \quad = \frac{u_{1} u_{2}}{AB(u_{1})\Gamma u_{1}} \int _{0}^{t} s^{u_{2}-1}(t-s)^{u_{1}-1}|Q_{4}(s, I_{h}(s)-Q_{4}\bigl(s, I_{h}^{*}(s) \bigr) \biggl\vert \,ds \\& \qquad {}+\frac{u_{2}(1-u_{1})t^{u_{2}-1}}{AB(u_{1})} \biggr\vert Q_{4}(t, I_{h}(t)-Q_{4} \bigl(t, I_{h}^{*}(t)\bigr)|\,ds \\& \quad \leq \biggl(\frac{u_{1} u_{2}}{AB(u_{1})\Gamma u_{1}} \int _{0}^{t} s^{u_{2}-1}(t-s)^{u_{1}-1}+ \frac{u_{2}(1-u_{1})t^{u_{2}-1}}{AB(u_{1})} \biggr)\phi _{4} \bigl\Vert I_{h}-I_{h}^{*} \bigr\Vert \\& \quad \leq \biggl( \frac{u_{1} u_{2}\Gamma u_{2}}{AB(u_{1}\Gamma (u_{1}+u_{2}))}+ \frac{u_{2}(1-u_{1})}{AB(u_{1})} \biggr)\phi _{4} \bigl\Vert I_{h}-I_{h}^{*} \bigr\Vert . \end{aligned}$$

Let $\alpha _{4}= ( \frac{u_{1} u_{2}\Gamma u_{2}}{AB(u_{1}\Gamma (u_{1}+u_{2}))}+ \frac{u_{2}(1-u_{1})}{AB(u_{1})} )\|I_{h}-I_{h}^{*}\|$, $\Delta _{4}=\phi _{4}$, so the above inequality becomes $|I_{h}-I_{h}^{*}|\leq \alpha _{4} \Delta _{4}$.

$$\begin{aligned}& \bigl\vert A(t)-A^{*}(t) \bigr\vert \\& \quad = \biggl\vert \biggl( \frac{u_{1} u_{2}}{AB(u_{1})\Gamma u_{1}} \int _{0}^{t} s^{u_{2}-1}(t-s)^{u_{1}-1}Q_{5} \bigl(s,A(s)\bigr)\,ds+ \frac{u_{2}(1-u_{1})t^{u_{2}-1}}{AB(u_{1})}Q_{5}\bigl(t,A(t)\bigr) \biggr) \\& \qquad {}- \biggl(\frac{u_{1} u_{2}}{AB(u_{1})\Gamma u_{1}} \int _{0}^{t} s^{u_{2}-1}(t-s)^{u_{1}-1}Q_{5} \bigl(s,A^{*}(s)\bigr)\,ds \\& \qquad {}+ \frac{u_{2}(1-u_{1})t^{u_{2}-1}}{AB(u_{1})}Q_{5} \bigl(t,A^{*}(t)\bigr) \biggr) \biggr\vert \\& \quad = \frac{u_{1} u_{2}}{AB(u_{1})\Gamma u_{1}} \int _{0}^{t} s^{u_{2}-1}(t-s)^{u_{1}-1} \bigl\vert Q_{5}(s, A(s)-Q_{5}\bigl(s, A^{*}(s) \bigr) \bigr\vert \,ds \\& \qquad {}+\frac{u_{2}(1-u_{1})t^{u_{2}-1}}{AB(u_{1})} \bigl\vert Q_{5}(t, A(t)-Q_{5} \bigl(t, A^{*}(t)\bigr) \bigr\vert \,ds \\& \quad \leq \biggl(\frac{u_{1} u_{2}}{AB(u_{1})\Gamma u_{1}} \int _{0}^{t} s^{u_{2}-1}(t-s)^{u_{1}-1}+ \frac{u_{2}(1-u_{1})t^{u_{2}-1}}{AB(u_{1})} \biggr)\phi _{5} \bigl\Vert A-A^{*} \bigr\Vert \\& \quad \leq \biggl( \frac{u_{1} u_{2}\Gamma u_{2}}{AB(u_{1}\Gamma (u_{1}+u_{2}))}+ \frac{u_{2}(1-u_{1})}{AB(u_{1})} \biggr)\phi _{5} \bigl\Vert A-A^{*} \bigr\Vert . \end{aligned}$$

Let $\alpha _{5}= ( \frac{u_{1} u_{2}\Gamma u_{2}}{AB(u_{1}\Gamma (u_{1}+u_{2}))}+ \frac{u_{2}(1-u_{1})}{AB(u_{1})} )\|A-A^{*}\|$, $\Delta _{5}=\phi _{5}$, so the above inequality becomes $|A-A^{*}|\leq \alpha _{5} \Delta _{5}$.

$$\begin{aligned}& \bigl\vert R(t)-R^{*}(t) \bigr\vert \\& \quad = \biggl\vert \biggl( \frac{u_{1} u_{2}}{AB(u_{1})\Gamma u_{1}} \int _{0}^{t} s^{u_{2}-1}(t-s)^{u_{1}-1}Q_{6} \bigl(s,R(s)\bigr)\,ds \\& \qquad {}+ \frac{u_{2}(1-u_{1})t^{u_{2}-1}}{AB(u_{1})}Q_{6}\bigl(t,R(t)\bigr) \biggr) \\& \qquad {}- \biggl(\frac{u_{1} u_{2}}{AB(u_{1})\Gamma u_{1}} \int _{0}^{t} s^{u_{2}-1}(t-s)^{u_{1}-1}Q_{6} \bigl(s,R^{*}(s)\bigr)\,ds \\& \qquad {}+ \frac{u_{2}(1-u_{1})t^{u_{2}-1}}{AB(u_{1})}Q_{6} \bigl(t,R^{*}(t)\bigr) \biggr) \biggr\vert \\& \quad = \frac{u_{1} u_{2}}{AB(u_{1})\Gamma u_{1}} \int _{0}^{t} s^{u_{2}-1}(t-s)^{u_{1}-1} \bigl\vert Q_{6}(s, R(s)-Q_{6}\bigl(s, R^{*}(s) \bigr) \bigr\vert \,ds \\& \qquad {}+\frac{u_{2}(1-u_{1})t^{u_{2}-1}}{AB(u_{1})} \bigl\vert Q_{6}(t, R(t)-Q_{6} \bigl(t, R^{*}(t)\bigr) \bigr\vert \,ds \\& \quad \leq \biggl(\frac{u_{1} u_{2}}{AB(u_{1})\Gamma u_{1}} \int _{0}^{t} s^{u_{2}-1}(t-s)^{u_{1}-1}+ \frac{u_{2}(1-u_{1})t^{u_{2}-1}}{AB(u_{1})} \biggr)\phi _{6} \bigl\Vert R-R^{*} \bigr\Vert \\& \quad \leq \biggl( \frac{u_{1} u_{2}\Gamma u_{2}}{AB(u_{1}\Gamma (u_{1}+u_{2}))}+ \frac{u_{2}(1-u_{1})}{AB(u_{1})} \biggr)\phi _{6} \bigl\Vert R-R^{*} \bigr\Vert . \end{aligned}$$

Let $\alpha _{6}= ( \frac{u_{1} u_{2}\Gamma u_{2}}{AB(u_{1}\Gamma (u_{1}+u_{2}))}+ \frac{u_{2}(1-u_{1})}{AB(u_{1})} )\|R-R^{*}\|$, $\Delta _{6}=\phi _{6}$, so the above inequality becomes $|R-R^{*}|\leq \alpha _{6} \Delta _{6}$. Consequently, by definition the fractal fractional model (1) is Hyers–Ulam stable. This completes the proof. □

## Numerical scheme

Numerical scheme for the fractal fractional order SE(Is)(Ih)AR epidemic model.

### Definition 3.1

Suppose that $\psi (t)$ is continuous and fractal differentiable on the interval $(u, v)$ with order $\Upsilon _{2}$, then the fractal fractional derivative of $\psi (t)$ with order $\Upsilon _{1}$ in the Riemann–Liouville sense having power law type kernel is given by

$$ {}^{\mathrm{FFP}}{}_{0}D^{\Upsilon _{1},\Upsilon _{2}}_{t}\psi (t)= \frac{1}{\Gamma (p-\Upsilon _{1}}\frac{d}{dt^{\Upsilon _{2}}} \int _{0}^{t} (t-s)^{p-\Upsilon _{1}-1}\psi (s)\,ds, $$

where $p-1< \Upsilon _{1}$, $\Upsilon _{2} \leq p \in \mathcal{N}$, and $\frac{d}{dt^{\Upsilon _{2}}}=\lim_{t\rightarrow s} \frac{\psi (t)-\psi (s)}{t^{\Upsilon _{2}}-s^{\Upsilon _{2}}}$.

### Definition 3.2

Suppose that $\psi (t)$ is continuous on the interval $(u, v)$, then the fractal fractional integral of $\psi (t)$ with order $\Upsilon _{1}$ having Mittag-Leffler type kernel is given by

$$\begin{aligned} {}^{\mathrm{FFM}}{}_{0}I^{\Upsilon _{1},\Upsilon _{2}}_{t}\psi (t) =& \frac{\Upsilon _{1} \Upsilon _{2}}{AB(\Upsilon _{1})\Gamma \Upsilon _{1}} \int _{0}^{t} s^{\Upsilon _{2}-1}\psi (s) (t-s)^{\Upsilon _{1}-1}\,ds \\ &{}+ \frac{\Upsilon _{2}(1-\Upsilon _{1})t^{(}\Upsilon _{2}-1)}{AB(\Upsilon _{1})} \psi (t). \end{aligned}$$

Let us consider ${}^{\mathrm{FFM}}{}_{0}D^{\Upsilon _{1},\Upsilon _{2}}_{t}\eta (t)= \mathcal{H}(t, \eta (t))R$, where $\eta (0)=\eta _{0}$ The above equation can be written in fractal fractional derivative as follows:

$${}^{\mathrm{FFR}}{}_{0}D^{\Upsilon _{1}}_{t}\eta (t)=\Upsilon _{2} t^{ \Upsilon _{2}-1}\mathcal{L}\bigl(t, \eta (t)\bigr)=\mathcal{H}\bigl(t, \eta (t)\bigr). $$

With the help of integral, we get

$$\begin{aligned} \eta (t) =&\eta (0)+\frac{1-\Upsilon _{1}}{AB(\Upsilon _{1})} \mathcal{H}\bigl(t, \eta (t)\bigr) \\ &{}+ \frac{\Upsilon _{1}}{AB(\Upsilon _{1})\Gamma \Upsilon _{1}} \int _{0}^{t} \zeta ^{\Upsilon _{2}-1}(t-\zeta )^{\Upsilon _{1}-1}\mathcal{H}\bigl( \zeta , \eta (\zeta )\bigr)\,d\zeta . \end{aligned}$$

Replacing $(t)$ with $t_{n+1}$, we have

9 $$\begin{aligned} \eta ^{n+1} =&\eta (0)+\frac{1-\Upsilon _{1}}{AB(\Upsilon _{1})} \mathcal{H} \bigl(t_{n}, \eta (t_{n})\bigr) \\ &{}+ \frac{\Upsilon _{1}}{AB(\Upsilon _{1})\Gamma \Upsilon _{1}} \int _{0}^{t^{n+1}} \zeta ^{\Upsilon _{2}-1}(t_{n+1}- \zeta )^{\Upsilon _{1}-1}\mathcal{H}\bigl( \zeta , \eta (\zeta )\bigr)\,d\zeta . \end{aligned}$$

By applying two-step Lagrange polynomial, we obtain

$$\begin{aligned} \theta \bigl(y, \eta (y)\bigr) =&\frac{(y-t_{k-1})H(t_{k}, \eta (t_{k}))}{t_{k}-t_{k-1}}- \frac{(y-t_{k})H(t_{k-1}, \eta (t_{k-1}))}{t_{k}-t_{k-1}} \\ =&\frac{H(t_{k}, \eta (t_{k})(y-t_{k-1})}{t_{k}-t_{k-1}}- \frac{H(t_{k-1}, \eta (t_{k-1}))(y-t_{k})}{t_{k}-t_{k-1}} \\ =&\frac{H(t_{k}, \eta _{k})(y-t_{k-1})}{h}- \frac{H(t_{k-1}, \eta _{k-1})(y-t_{k})}{h}. \end{aligned}$$

Applying Lagrange polynomial to equation (9), we get

$$\begin{aligned} \eta ^{n+1} =&\eta (0)+\frac{1-\Upsilon _{1}}{AB(\Upsilon _{1})} \mathcal{H} \bigl(t_{n}, \eta (t_{n})\bigr) \\ &{}+ \frac{\Upsilon _{1}}{AB(\Upsilon _{1})\Gamma \theta _{1}}\sum _{i=1}^{n} \biggl[\frac{\mathcal{H}(t_{i}, \eta (t_{i}))}{h} \int _{t_{k}}^{t^{k+1}} (\zeta -t_{i-1}) (t_{n+1}-\zeta )^{\Upsilon _{1}-1}\,d\zeta \\ &{}-\frac{\mathcal{H}(t_{i-1}, \eta (t_{i-1}))}{h} \int _{t_{k}}^{t^{n+1}} (\zeta -t_{i}) (t_{n+1}-\zeta )^{\Upsilon _{1}-1}\,d\zeta \biggr]. \end{aligned}$$

Now, solving the integral, we get

$$\begin{aligned} \eta ^{n+1} =&\eta (0)+\frac{1-\Upsilon _{1}}{AB(\Upsilon _{1})} \mathcal{H} \bigl(t_{n}, \eta (t_{n})\bigr) \\ &{}+\frac{\Upsilon _{1} h^{\Upsilon _{1}}}{\Gamma (\Upsilon _{1}+2)} \sum _{i=1}^{n} \bigl[{\mathcal{H} \bigl(t_{i}, \eta (t_{i})\bigr)} \bigl((n+1-i)^{\Upsilon }_{1}(n-i+2+ \Upsilon _{1}) \\ &{}-(n-i)^{\Upsilon }_{1}(n-i+2+2 \Upsilon _{1}) \bigr) \\ &{}-\mathcal{H}(t_{i-1}, \eta _{i-1}) \bigl((n+1-i)^{\Upsilon _{1}+1}-(n-i+1+ \Upsilon _{1}) (n-i)^{\Upsilon _{1}} \bigr) \bigr]. \end{aligned}$$

Replacing the value of $\mathcal{H}(t, \eta (t))$, we have

$$\begin{aligned} \eta ^{n+1} =&\eta (0)+\Upsilon _{2} t^{\Upsilon _{2}-1} \frac{1-\Upsilon _{1}}{AB(\Upsilon _{1})}\mathcal{I}\bigl(t_{n}, \eta (t_{n})\bigr) \\ &{}+\Upsilon _{2} t^{\Upsilon _{2}-1} \frac{\Upsilon _{2} h^{\Upsilon _{1}}}{\Gamma (\Upsilon _{1}+2)} \sum _{i=1}^{n} \bigl[{\mathcal{I}\bigl(t_{i}, \eta (t_{i})\bigr)} \bigl((n+1-i)^{\Upsilon }_{1}(n-i+2+ \Upsilon _{1}) \\ &{}-(n-i)^{\Upsilon }_{1}(n-i+2+2 \Upsilon _{1}) \bigr) \\ &{}-\mathcal{I}(t_{i-1}, \eta _{i-1}) \bigl((n+1-i)^{\Upsilon _{1}+1}-(n-i+1+ \Upsilon _{1}) (n-i)^{\Upsilon _{1}} \bigr) \bigr]. \end{aligned}$$

Now the system of equations with kernels $\mathcal{H}_{i}$, $i\in \mathcal{N}_{1}^{6}$ with initial conditions $S(0) = E(0) = I_{s}(0) = I_{h}(0) = A(0) = R(0) = 0$:

$$\begin{aligned}& S(t) = S(0)+ \frac{\Upsilon _{1} \Upsilon _{2}}{AB(\Upsilon _{1})\Gamma \Upsilon _{1}} \int _{0}^{t} s^{\Upsilon _{2}-1}(t-s)^{\Upsilon _{1}-1}H_{1} \bigl(s,S(s)\bigr)\,ds \\& \hphantom{S(t) =} {}+\frac{\Upsilon _{2}(1-\Upsilon _{1})t^{\Upsilon _{2}-1}}{AB(\Upsilon _{1})}H_{1}\bigl(t,S(t)\bigr), \\& E(t) = E(0)+ \frac{\Upsilon _{1} \Upsilon _{2}}{AB(\Upsilon _{1})\Gamma \Upsilon _{1}} \int _{0}^{t} s^{\Upsilon _{2}-1}(t-s)^{\Upsilon _{1}-1}H_{2} \bigl(s,E(s)\bigr)\,ds \\& \hphantom{E(t) =} {}+\frac{\Upsilon _{2}(1-\Upsilon _{1})t^{\Upsilon _{2}-1}}{AB(\Upsilon _{1})}H_{2}\bigl(t,E(t)\bigr), \\& I_{s}(t) = I_{s}(0)+ \frac{\Upsilon _{1} \Upsilon _{2}}{AB(\Upsilon _{1})\Gamma \Upsilon _{1}} \int _{0}^{t} s^{\Upsilon _{2}-1}(t-s)^{\Upsilon _{1}-1}H_{3} \bigl(s,I_{s}(s)\bigr)\,ds \\& \hphantom{I_{s}(t) =} {}+\frac{\Upsilon _{2}(1-\Upsilon _{1})t^{\Upsilon _{2}-1}}{AB(\Upsilon _{1})}H_{3}\bigl(t,I_{s}(t)\bigr), \\& I_{h}(t) = I_{h}(0)+ \frac{\Upsilon _{1} \Upsilon _{2}}{AB(\Upsilon _{1})\Gamma \Upsilon _{1}} \int _{0}^{t} s^{\Upsilon _{2}-1}(t-s)^{\Upsilon _{1}-1}H_{4} \bigl(s,I_{h}(s)\bigr)\,ds \\& \hphantom{I_{h}(t) =} {}+\frac{\Upsilon _{2}(1-\Upsilon _{1})t^{\Upsilon _{2}-1}}{AB(\Upsilon _{1})}H_{4}\bigl(t,I_{h}(t)\bigr), \\& A(t) = A(0)+ \frac{\Upsilon _{1} \Upsilon _{2}}{AB(\Upsilon _{1})\Gamma \Upsilon _{1}} \int _{0}^{t} s^{\Upsilon _{2}-1}(t-s)^{\Upsilon _{1}-1}H_{5} \bigl(s,A(s)\bigr)\,ds \\& \hphantom{A(t) =} {}+\frac{\Upsilon _{2}(1-\Upsilon _{1})t^{\Upsilon _{2}-1}}{AB(\Upsilon _{1})}H_{5}\bigl(t,A(t)\bigr), \\& R(t) = R(0)+ \frac{\Upsilon _{1} \Upsilon _{2}}{AB(\Upsilon _{1})\Gamma \Upsilon _{1}} \int _{0}^{t} s^{\Upsilon _{2}-1}(t-s)^{\Upsilon _{1}-1}H_{6} \bigl(s,R(s)\bigr)\,ds \\& \hphantom{R(t) =} {}+\frac{\Upsilon _{2}(1-\Upsilon _{1})t^{\Upsilon _{2}-1}}{AB(\Upsilon _{1})}H_{6}\bigl(t,R(t)\bigr). \end{aligned}$$

Now from the numerical scheme for fractal-fractional order model (1), we have

$$\begin{aligned}& S_{n+1} = \eta (0)+\Upsilon _{2} t^{\Upsilon _{2}-1} \frac{1-\Upsilon _{1}}{AB(\Upsilon _{1})}{F_{1}}\bigl(t_{n}, S(t_{n}) \bigr)+ \Upsilon _{2} t^{\Upsilon _{2}-1} \frac{\Upsilon _{2} h^{\Upsilon _{1}}}{\Gamma (\Upsilon _{1}+2)} \\& \hphantom{S_{n+1} = }{} \times \sum _{i=1}^{n} \bigl[{{F_{1}} \bigl(t_{i}, S(t_{i})\bigr)} \bigl((n+1-i)^{\Upsilon }_{1}(n-i+2+ \Upsilon _{1}) \\& \hphantom{S_{n+1} = } {}-(n-i)^{\Upsilon }_{1}(n-i+2+2 \Upsilon _{1}) \bigr) \\& \hphantom{S_{n+1} = } {}-{F_{1}}(t_{i-1}, S_{i-1}) \bigl((n+1-i)^{\Upsilon _{1}+1}-(n-i+1+ \Upsilon _{1}) (n-i)^{\Upsilon _{1}} \bigr) \bigr], \\& E_{n+1} = \eta (0)+\Upsilon _{2} t^{\Upsilon _{2}-1} \frac{1-\Upsilon _{1}}{AB(\Upsilon _{1})}{F_{2}}\bigl(t_{n}, E(t_{n}) \bigr)+ \Upsilon _{2} t^{\Upsilon _{2}-1} \frac{\Upsilon _{2} h^{\Upsilon _{1}}}{\Gamma (\Upsilon _{1}+2)} \\& \hphantom{E_{n+1} =}{} \times \sum _{i=1}^{n} \bigl[{F_{2}} \bigl(t_{i}, E(t_{i})\bigr) \bigl((n+1-i)^{\Upsilon }_{1}(n-i+2+ \Upsilon _{1}) \\& \hphantom{E_{n+1} =}{}-(n-i)^{\Upsilon }_{1}(n-i+2+2 \Upsilon _{1}) \bigr) \\& \hphantom{E_{n+1} =} {}- F_{2}(t_{i-1}, E_{i-1}) \bigl((n+1-i)^{\Upsilon _{1}+1}-(n-i+1+ \Upsilon _{1}) (n-i)^{\Upsilon _{1}} \bigr) \bigr], \\& I_{s_{n+1}} = \eta (0)+\Upsilon _{2} t^{\Upsilon _{2}-1} \frac{1-\Upsilon _{1}}{AB(\Upsilon _{1})}{F_{3}}\bigl(t_{n}, I_{s}(t_{n}) \bigr)+ \Upsilon _{2} t^{\Upsilon _{2}-1} \frac{\Upsilon _{2} h^{\Upsilon _{1}}}{\Gamma (\Upsilon _{1}+2)} \\& \hphantom{I_{s_{n+1}} =}{} \times \sum _{i=1}^{n} \bigl[{F_{3}} \bigl(t_{i}, I_{s}(t_{i})\bigr) \bigl((n+1-i)^{\Upsilon }_{1}(n-i+2+\Upsilon _{1})-(n-i)^{\Upsilon }_{1}(n-i+2+2 \Upsilon _{1}) \bigr) \\& \hphantom{I_{s_{n+1}} =}{}- {F_{3}}(t_{i-1}, I_{s_{i-1}}) \bigl((n+1-i)^{\Upsilon _{1}+1}-(n-i+1+ \Upsilon _{1}) (n-i)^{\Upsilon _{1}} \bigr) \bigr], \\& I_{h_{n+1}} = \eta (0)+\Upsilon _{2} t^{\Upsilon _{2}-1} \frac{1-\Upsilon _{1}}{AB(\Upsilon _{1})}{F_{4}}\bigl(t_{n}, I_{h}(t_{n}) \bigr)+ \Upsilon _{2} t^{\Upsilon _{2}-1} \frac{\Upsilon _{2} h^{\Upsilon _{1}}}{\Gamma (\Upsilon _{1}+2)} \\& \hphantom{I_{h_{n+1}} =}{}\times \sum _{i=1}^{n} \bigl[{F_{4}} \bigl(t_{i}, I_{h}(t_{i})\bigr) \bigl((n+1-i)^{\Upsilon }_{1}(n-i+2+\Upsilon _{1})-(n-i)^{\Upsilon }_{1}(n-i+2+2 \Upsilon _{1}) \bigr) \\& \hphantom{I_{h_{n+1}} =} {}- {F_{4}}(t_{i-1}, I_{h}{i-1}) \bigl((n+1-i)^{\Upsilon _{1}+1}-(n-i+1+ \Upsilon _{1}) (n-i)^{\Upsilon _{1}} \bigr) \bigr], \\& A_{n+1} = \eta (0)+\Upsilon _{2} t^{\Upsilon _{2}-1} \frac{1-\Upsilon _{1}}{AB(\Upsilon _{1})}{F_{5}}\bigl(t_{n}, A(t_{n}) \bigr)+ \Upsilon _{2} t^{\Upsilon _{2}-1} \frac{\Upsilon _{2} h^{\Upsilon _{1}}}{\Gamma (\Upsilon _{1}+2)} \\& \hphantom{A_{n+1} =}{} \times \sum _{i=1}^{n} \bigl[{F_{5}} \bigl(t_{i}, A(t_{i})\bigr) \bigl((n+1-i)^{\Upsilon }_{1}(n-i+2+ \Upsilon _{1})-(n-i)^{\Upsilon }_{1}(n-i+2+2 \Upsilon _{1}) \bigr) \\& \hphantom{A_{n+1} =} {}- {F_{5}}(t_{i-1}, A_{i-1}) \bigl((n+1-i)^{\Upsilon _{1}+1}-(n-i+1+ \Upsilon _{1}) (n-i)^{\Upsilon _{1}} \bigr) \bigr], \\& R_{n+1} = \eta (0)+\Upsilon _{2} t^{\Upsilon _{2}-1} \frac{1-\Upsilon _{1}}{AB(\Upsilon _{1})}{F_{6}}\bigl(t_{n}, R(t_{n}) \bigr)+ \Upsilon _{2} t^{\Upsilon _{2}-1} \frac{\Upsilon _{2} h^{\Upsilon _{1}}}{\Gamma (\Upsilon _{1}+2)} \\& \hphantom{R_{n+1} =}{} \times \sum _{i=1}^{n} \bigl[{F_{6}} \bigl(t_{i}, R(t_{i})\bigr) \bigl((n+1-i)^{\Upsilon }_{1}(n-i+2+ \Upsilon _{1}) \\& \hphantom{R_{n+1} =} {}-(n-i)^{\Upsilon }_{1}(n-i+2+2 \Upsilon _{1}) \bigr) \\& \hphantom{R_{n+1} =} {}-{F_{6}}(t_{i-1}, R_{i-1}) \bigl((n+1-i)^{\Upsilon _{1}+1}-(n-i+1+ \Upsilon _{1}) (n-i)^{\Upsilon _{1}} \bigr) \bigr]. \end{aligned}$$

### Computational results

Here, we present the computational results based on the literature. The initial values for the population are: $S(0)=6{,}778{,}382$, $E(0)=1$, $I_{s}(0)=0$, $I_{h}(0)=0$, $A(0)=0$, $R(0)=0$, and the parametric values are: $b_{1}=57{,}554$, $b_{2}=1/85$, $N=6{,}778{,}383$, $\beta =1/N$, $\beta _{a}r=1$, $\beta _{h}r=1/80$, $\gamma =1/5.5$, $\eta =0$, $\alpha =0.12$, $\tau _{0}=1/10$, $p_{s}=0.55$, $p_{h}=0.20$, $k_{v}=0.001$, $k_{T}=0.004$ [37].

**Figure 1:**
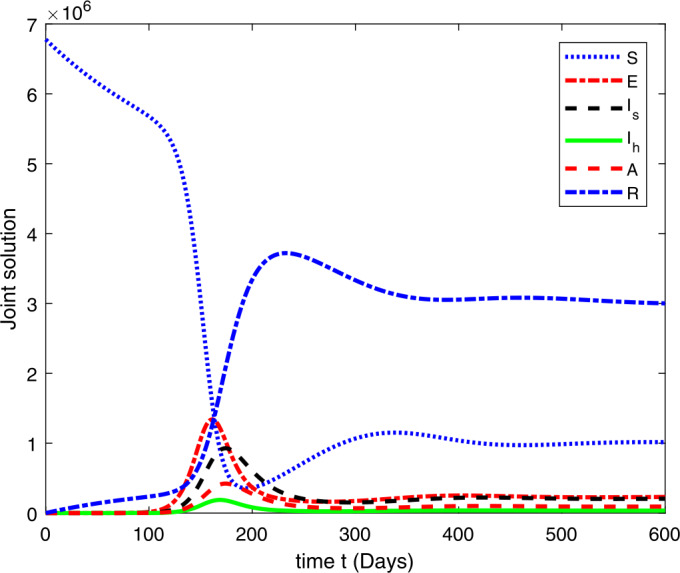
Joint solution of COVID 19 model (1) for the order 1.0

**Figure 2:**
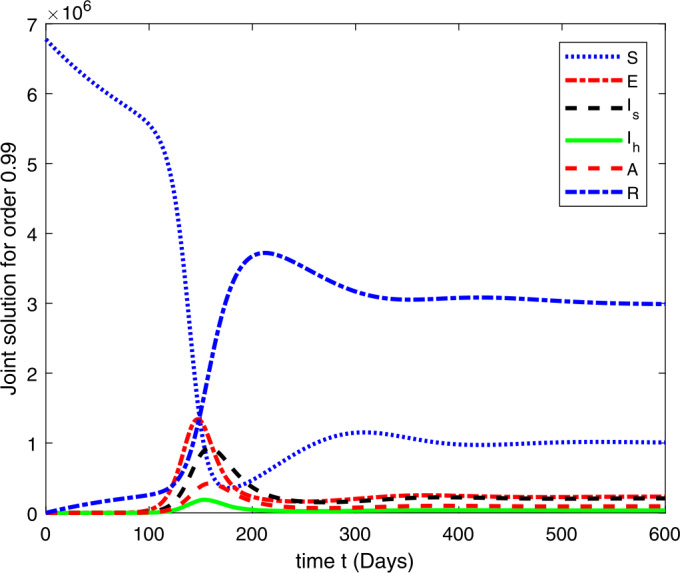
Joint solution of COVID 19 model (1) for the order 0.99

**Figure 3:**
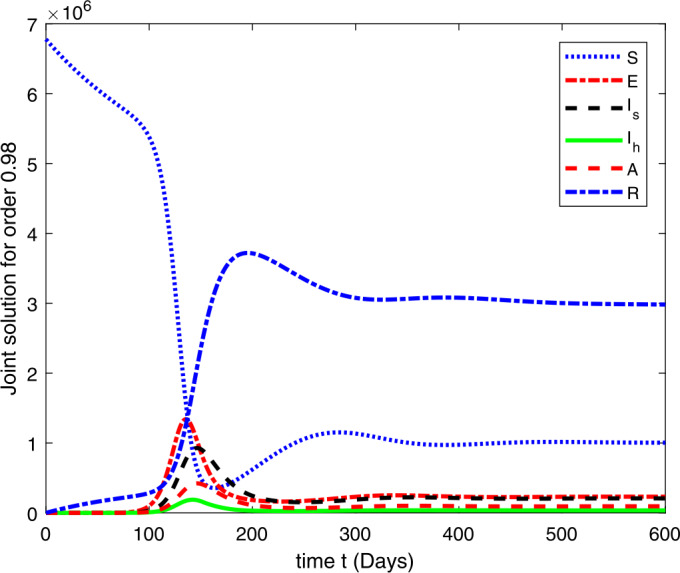
Joint solution of COVID 19 model (1) for the order 0.98

**Figure 4:**
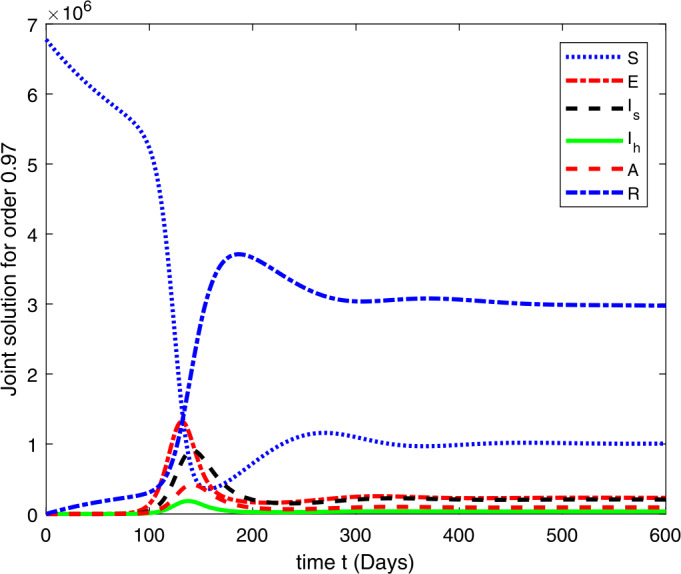
Joint solution of COVID 19 model (1) for the order 0.97

**Figure 5:**
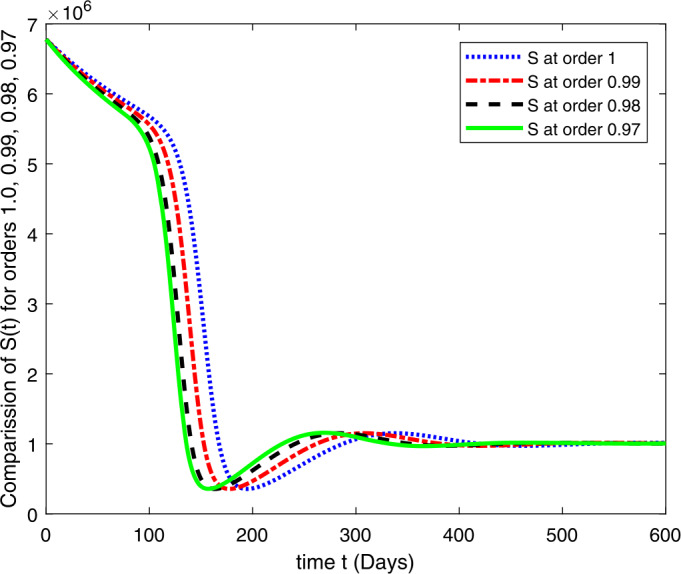
Computational results for $S(t)$ while the fractional orders are 1.0, 0.99, 0.98, 0.97

**Figure 6:**
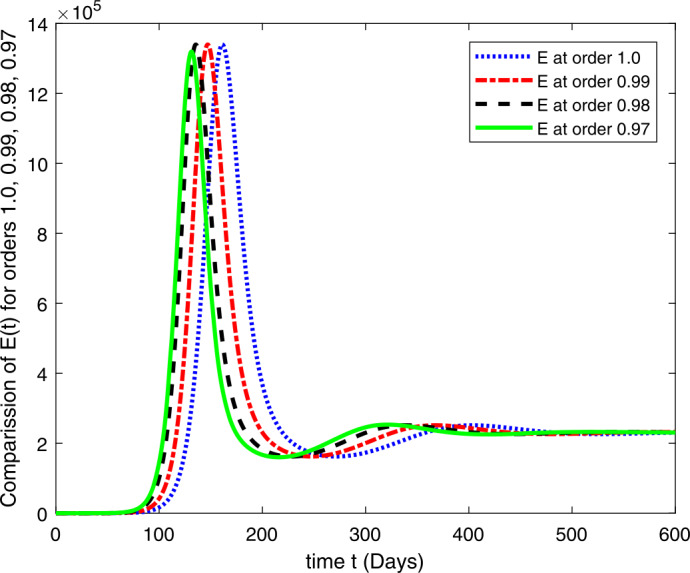
A comparative analysis of $E(t)$ for the fractional orders 1.0, 0.99, 0.98, 0.97

**Figure 7:**
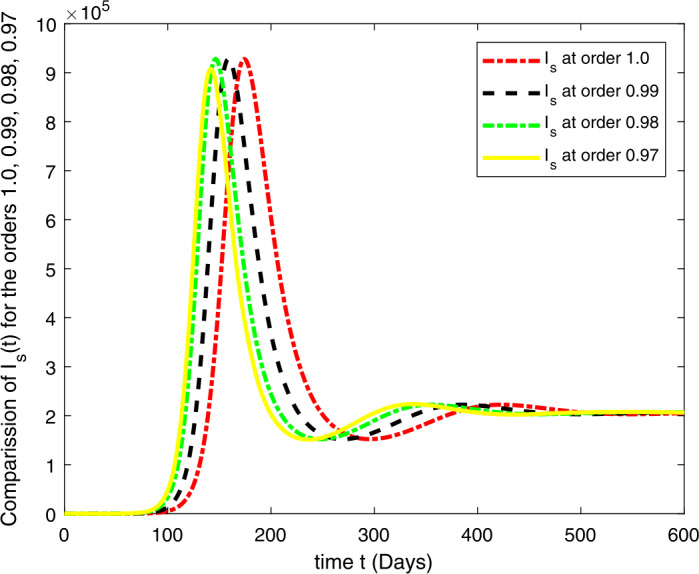
Computational results for $I_{s}(t)$ for the fractional orders 1.0, 0.99, 0.98, 0.97

**Figure 8:**
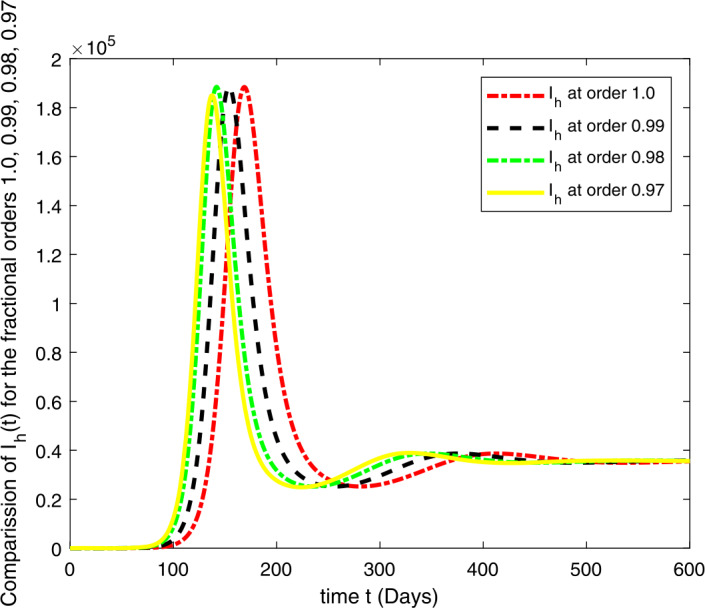
A comparative analysis of $I_{h}(t)$ for the fractional orders 1.0, 0.99, 0.98, 0.97

**Figure 9:**
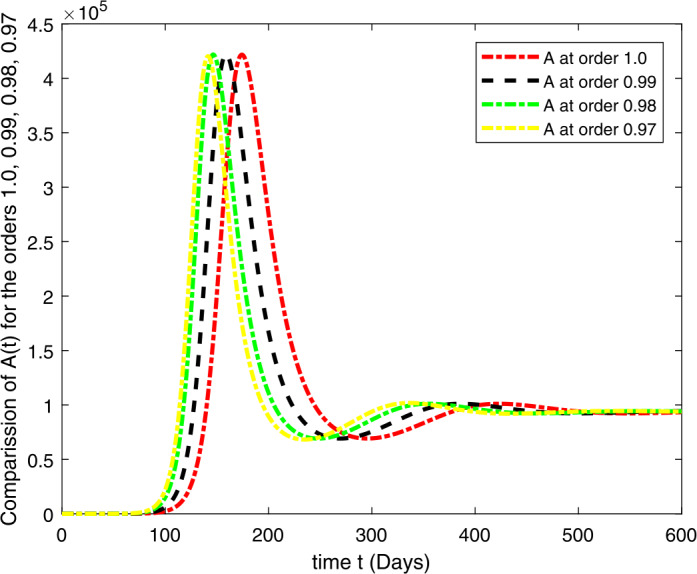
A comparative analysis of $A(t)$ for the fractional orders 1.0, 0.99, 0.98, 0.97

**Figure 10:**
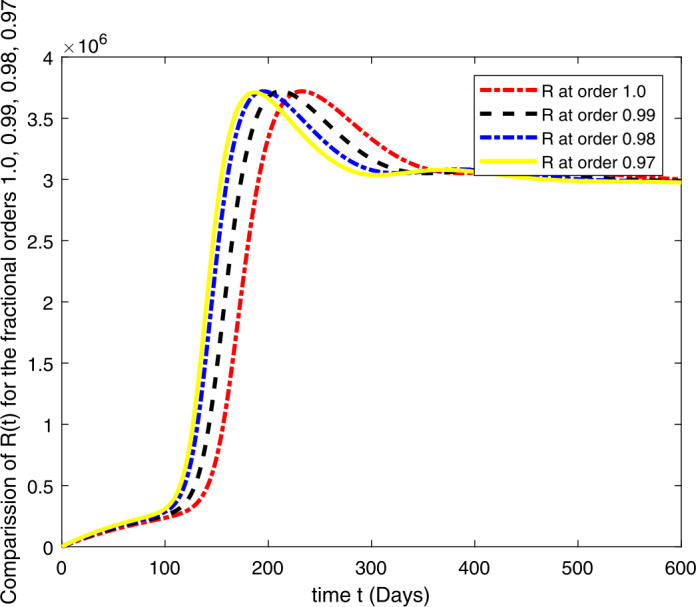
A comparative analysis of $R(t)$ for the fractional orders 1.0, 0.99, 0.98, 0.97

The computational results are given via ten graphs. In the Fig. 1, we have given the numerical solution of the suggested model for order 1 which is compared with the numerical solution of the model for orders 0.99, 0.98, 0.97 in Figs. 2, 3 and 4, respectively. Figure 5 is for the comparative study of $S(t)$ for the different orders. Similarly, $E(t)$, $I_{s}(t)$, $I_{h}(t)$, $A(t)$ and $R(t)$ are analysed for different fractional orders in the Figs. 6, 7, 8, 9, and 10 respectively.

## Conclusion

In current manuscript, we have established a detailed analysis related to the results about existence and uniqueness results with Ulam stability. The subjective problem is nonlocal multipoint BVPs involving delay term of FDEs. The respective analysis has been established via using classical fixed point theory and some results of nonlinear functional analysis. The whole analysis has been demonstrated via computational results based on the literature. The initial values for the population are: $S(0)=6{,}778{,}382$, $E(0)=1$, $I_{s}(0)=0$, $I_{h}(0)=0$, $A(0)=0$, $R(0)=0$, and the parametric values are: $b_{1}=57{,}554$, $b_{2}=1/85$, $N=6{,}778{,}383$, $\beta =1/N$, $\beta _{a}r=1$, $\beta _{h}r=1/80$, $\gamma =1/5.5$, $\eta =0$, $\alpha =0.12$, $\tau _{0}=1/10$, $p_{s}=0.55$, $p_{h}=0.20$, $k_{v}=0.001$, $k_{T}=0.004$ [37]. The results are more realistic and of the same behavior as the classical ones. Our results are getting more similar to the integer order ones for the orders closer to 1.0.

## Data Availability

No data were used to support this study.

## References

[1] Lu H., Stratton C.W., Tang Y.W. (2020). Outbreak of pneumonia of unknown etiology in Wuhan China: the mystery and the miracle. J. Med. Virol..

[2] Zimmer, C., Corum, J., Wee, S.-L.: Coronavirus Vaccine Tracker (2020) https://www.nytimes.com/interactive/2020/science/coronavirus-vaccine-tracker.html

[3] CDC December: (2020) https://www.cdc.gov/coronavirus/2019-ncov/vaccines/different-vaccines.html

[4] Goyal M., Baskonus H.M., Prakash A. (2019). An efficient technique for a time fractional model of Lassa hemorrhagic fever spreading in pregnant women. Eur. Phys. J. Plus.

[5] Gao W., Veeresha P., Prakasha D.G., Baskonus H.M., Yel G. (2020). New approach for the model describing the deathly disease in pregnant women using Mittag-Leffler function. Chaos Solitons Fractals.

[6] Kumar D., Singh J., Al-Qurashi M., Baleanu D. (2019). A new fractional SIRS-SI malaria disease model with application of vaccines, anti-malarial drugs, and spraying. Adv. Differ. Equ..

[7] Shah K., Alqudah M.A., Jarad F., Abdeljawad T. (2020). Semi-analytical study of Pine Wilt disease model with convex rate under Caputo-Fabrizio fractional order derivative. Chaos Solitons Fractals.

[8] Pang J., Cui J.A., Zhou X. (2010). Dynamical behavior of a Hepatitis B virus transmission model with vaccination. J. Theor. Biol..

[9] Zou L., Zhang W., Ruan S. (2010). Modeling the transmission dynamics and control of Hepatitis B virus in China. J. Theor. Biol..

[10] Chen T.-M., Rui J., Wang Q.-P., Zhao Z.-Y., Cui J.-A., Yin L. (2020). A mathematical model for simulating the phase-based transmissibility of a novel coronavirus. Infect. Dis. Poverty.

[11] Zhou P., Yang X., Wang X. (2020). A pneumonia outbreak associated with a new coronavirus of probable bat origin. Nature.

[12] Li Q., Guan X., Wu P. (2020). Early transmission dynamics in Wuhan, China, of novel coronavirus 221 infected pneumonia. N. Engl. J. Med..

[13] Huang C., Wang Y. (2020). Clinical features of patients infected with 2019 novel coronavirus in Wuhan China. Lancet.

[14] Podlubny I. (1999). Fractional Differential Equations, Mathematics in Science and Engineering.

[15] Lakshmikantham V., Leela S., Vasundhara J. (2009). Theory of Fractional Dynamic Systems.

[16] Rossikhin Y.A., Shitikova M.V. (1997). Applications of fractional calculus to dynamic problems of linear and nonlinear hereditary mechanics of solids. Appl. Mech. Rev..

[17] Machado J., Kiryakova V., Mainardi V. (2011). Recent history of fractional calculus. Commun. Nonlinear Sci. Numer. Simul..

[18] Baleanu D., Diethelm K., Scalas E., Trujillo J. (2012). Fractional Calculus Models and Numerical Methods.

[19] Heymans N., Bauwens J. (1994). Fractal rheological models and fractional differential equations for viscoelastic behaviour. Rheol. Acta.

[20] Mainardi F. (2010). Fractional Calculus and Waves in Linear Viscoelasticity.

[21] Machado J.T., Kiryakova V., Mainardi F. (2011). Recent history of fractional calculus. Commun. Nonlinear Sci. Numer. Simul..

[22] Loverro, A.: Fractional calculus: history, definitions and applications for the engineer. Rapport Technique, Univeristy of Notre Dame: Department of Aerospace and Mechanical Engineering, Dame (2004)

[23] Osler T.J. (1971). Fractional derivatives and Leibniz rule. Am. Math. Mon..

[24] Malinowska A.B., Odzijewicz T., Torres D.F. (2015). Advanced Methods in the Fractional Calculus of Variations.

[25] Kilbas A.A., Srivastava H.M., Trujillo J.J. (2003). Fractional differential equations: a emergent field in applied and mathematical sciences. Factorization, Singular Operators and Related Problems.

[26] Jumarie G. (2006). Modified Riemann Liouville derivative and fractional Taylor series of nondifferentiable functions further results. Comput. Math. Appl..

[27] Algahtani O.J. (2016). Comparing the Atangana Baleanu and Caputo Fabrizio derivative with fractional order: Allen Cahn model. Chaos Solitons Fractals.

[28] Kao Y., Gao C., Wang D. (2008). Global exponential stability of reaction-diffusion Hopfield neural networks with continuously distributed delays. J. Math. Anal. Appl..

[29] Luo J. (2008). Fixed points and exponential stability of mild solutions of stochastic partial differential equations with delays. J. Math. Anal. Appl..

[30] Wang L. (2017). Global well-posedness and stability of the mild solutions for a class of stochastic partial functional differential equations. Sci. China Math..

[31] Wang L., Gao Y. (2006). Global exponential robust stability of reaction diffusion interval neural networks with time-varying delays. Phys. Lett. A.

[32] Wang L., Zhang R., Wang Y. (2009). Global exponential stability of reaction-diffusion cellular neural networks with S-type distributed time delays. Nonlinear Anal., Real World Appl..

[33] Lu J.G. (2008). Global exponential stability and periodicity of reaction diffusion delayed recurrent neural networks with Dirichlet boundary conditions. Chaos Solitons Fractals.

[34] Atangana A., Akgul A., Owolabi K.M. (2020). Analysis of fractal fractional differential equations. Alex. Eng. J..

[35] Chen W. (2006). A speculative study of 2/3-order fractional Laplacian modeling of turbulence: some thoughts and conjectures. Chaos.

[36] Meerschaert M.M., Tadjeran C. (2004). Finite difference approximations for fractional advection dispersion flow equations. J. Comput. Appl. Math..

[37] Djomegni P.M., Haggar M.D., Adigo W.T. (2021). Mathematical model for Covid-19 with “protected susceptible” in the post-lockdown era. Alex. Eng. J..

[38] Alqahtani B., Aydi H., Karapinar E., Rakocevic V. (2019). A solution for Volterra fractional integral equations by hybrid contractions. Mathematics.

[39] Karapinar E., Fulga A., Rashid M., Shahid L., Aydi H. (2019). Large contractions on quasi-metric spaces with an application to nonlinear fractional differential equations. Mathematics.

[40] Baleanu D., Jajarmi A., Mohammadi H., Rezapour S. (2020). A new study on the mathematical modelling of human liver with Caputo–Fabrizio fractional derivative. Chaos Solitons Fractals.

[41] Tuan N.H., Mohammadi H., Rezapour S. (2020). A mathematical model for COVID-19 transmission by using the Caputo fractional derivative. Chaos Solitons Fractals.

[42] Mohammadi H., Kumar S., Rezapour S., Etemad S. (2021). A theoretical study of the Caputo–Fabrizio fractional modeling for hearing loss due to Mumps virus with optimal control. Chaos Solitons Fractals.

[43] Mohammadi, H., Rezapour, S., Jajarmi, A.: On the fractional SIRD mathematical model and control for the transmission of COVID-19: the first and the second waves of the disease in Iran and Japan. ISA Trans. (2021) 10.1016/j.isatra.2021.04.012 PMC8035661 33867134

[44] Baleanu D., Etemad S., Rezapour S. (2020). A hybrid Caputo fractional modeling for thermostat with hybrid boundary value conditions. Bound. Value Probl..

[45] Shaikh A.S., Shaikh I.N., Nisar K.S. (2020). A mathematical model of COVID-19 using fractional derivative: outbreak in India with dynamics of transmission and control. Adv. Differ. Equ..

[46] Singh H., Srivastava H.M., Hammouch Z., Nisar K.S. (2021). Numerical simulation and stability analysis for the fractional-order dynamics of COVID-19. Results Phys..

[47] Baba I.A., Yusuf A., Nisar K.S., Abdel-Aty A.H., Nofal T.A. (2021). Mathematical model to assess the imposition of lockdown during COVID-19 pandemic. Results Phys..

[48] Aghdaoui H., Alaoui A.L., Nisar K.S., Tilioua M. (2021). On analysis and optimal control of a SEIRI epidemic model with general incidence rate. Results Phys..

[49] Panda S.K., Ravichandran C., Hazarika B. (2021). Results on system of Atangana-Baleanu fractional order Willis aneurysm and nonlinear singularly perturbed boundary value problems. Chaos Solitons Fractals.

[50] Logeswari, K., Ravichandran, C., Nisar, K.S.: Mathematical model for spreading of COVID-19 virus with the Mittag-Leffler kernel. Numer. Methods Partial Differ. Equ. (2020) 10.1002/num.22652 PMC7753447 33362342

[51] Valliammal N., Ravichandran C., Nisar K.S. (2020). Solutions to fractional neutral delay differential nonlocal systems. Chaos Solitons Fractals.

